# Patient-, organization-, and system-level barriers and facilitators to preventive oral health care: a convergent mixed-methods study in primary dental care

**DOI:** 10.1186/s13012-015-0366-2

**Published:** 2016-01-12

**Authors:** Anna Rose Templeton, Linda Young, Alison Bish, Wendy Gnich, Heather Cassie, Shaun Treweek, Debbie Bonetti, Douglas Stirling, Lorna Macpherson, Sharon McCann, Jan Clarkson, Craig Ramsay, With the PMC study team

**Affiliations:** 1 University of Dundee, Dundee, UK; 2 NHS Education for Scotland, Edinburgh, UK; 3 University of Glasgow, Glasgow, UK; 4 University of Aberdeen, Aberdeen, UK

## Abstract

**Background:**

Dental caries is the most common chronic disease of adult and childhood, a largely preventable yet widespread, costly public health problem. This study identified patient-, organization-, and system-level factors influencing routine delivery of recommended care for prevention and management of caries in primary dental care.

**Methods:**

A convergent mixed-methods design assessed six guidance-recommended behaviours to prevent and manage caries (recording risk, risk-based recall intervals, applying fluoride varnish, placing preventive fissure sealants, demonstrating oral health maintenance, taking dental x-rays). A diagnostic questionnaire assessing current practice, beliefs, and practice characteristics was sent to a random sample of 651 dentists in National Health Service (NHS) Scotland. Eight in-depth case studies comprising observation of routine dental visits and dental team member interviews were conducted. Patient feedback was collected from adult patients with recent checkups at case study practices. Key informant interviews were conducted with decision makers in policy, funding, education, and regulation. The Theoretical Domains Framework within the Behaviour Change Wheel was used to identify and describe patient-, organization-, and system-level barriers and facilitators to care. Findings were merged into a matrix describing theoretical domains salient to each behaviour. The matrix and Behaviour Change Wheel were used to prioritize behaviours for change and plan relevant intervention strategies.

**Results:**

Theoretical domains associated with best practice were identified from the questionnaire (*N*-196), case studies (*N* = 8 practices, 29 interviews), and patient feedback (*N* = 19). Using the study matrix, key stakeholders identified priority behaviours (use of preventive fissure sealants among 6–12-year-olds) and strategies (audit and feedback, patient informational campaign) to improve guidance implementation. Proposed strategies were assessed as appropriate for immediate implementation and suitable for development with remaining behaviours.

**Conclusions:**

Specific, theoretically based, testable interventions to improve caries prevention and management were coproduced by patient-, practice-, and policy-level stakeholders. Findings emphasize duality of behavioural determinants as barriers and facilitators, patient influence on preventive care delivery, and benefits of integrating multi-level interests when planning interventions in a dynamic, resource-constrained environment. Interventions identified in this study are actively being used to support ongoing implementation initiatives including guidance, professional development, and oral health promotion.

**Electronic supplementary material:**

The online version of this article (doi:10.1186/s13012-015-0366-2) contains supplementary material, which is available to authorized users.

## Background

Despite being highly preventable, dental caries is a widespread and costly public health problem [1]. Globally, caries is the most common chronic disease of childhood and adulthood [1, 2]. Similar to other chronic diseases, caries aetiology is complex, influenced strongly by socio-economic factors in both childhood and adulthood [3–7]. Caries can have serious health sequelae and negatively impact quality of life and productivity across the lifespan [2, 3, 8–10]. In Scotland, despite continued improvements in national child caries rates, nearly half of primary 1 (4–7 years old) children (47 %) and a third (36 %) of primary 7 (10–13 years old) children in high-deprivation areas have obvious decay [11, 12]. Children with obvious decay have a higher burden of disease (decayed, missing, or filled teeth) and low rates of restoration: 14 % P1 [11] and 55 % P7 [12]. Current caries rates for adults in Scotland are unknown. The most recent data indicate that 26 % of the adult population had not attended a dental appointment in the past 2 years; in the most deprived areas, 32 % of adults had not seen a dentist in 2 years [13]. Within the rest of the UK, lower income children have similarly high caries prevalence (41 % of 5-year-olds, 59 % of 15-year-olds) [14] and only 53 % of adults had attended a dental appointment within the past 3 years [15].

Population- and clinical-level initiatives have been developed to promote oral health in Scotland. National guidance on oral health assessment in adults [16] and caries prevention and management in children [17, 18] has been published by the Scottish Dental Clinical Effectiveness Programme (SDCEP) and Scottish Intercollegiate Guidelines Network (SIGN). In an effort to shift practice toward preventive care, integrate community and clinical oral health prevention, and reduce health inequalities among children in Scotland, the Scottish Government has funded the development and implementation of the Childsmile programme [19]. Childsmile initiatives delivered in primary dental care include the promotion of dental clinic-based fluoride varnish application for all children over 2 years of age and enhanced oral health advice targeting high-risk children [20]. Despite these efforts, evaluations of the SDCEP child caries guidance and Childsmile programme reveal that the majority of dentists do not always follow key recommendations and implementation is typically influenced by numerous factors (e.g. knowledge, beliefs about consequences, professional roles and identity, social influences) which often act as both barriers and facilitators to care[21, 22].

Guidance implementation relies on strategies that consider the needs and experiences of users across multiple levels, the context and culture of care, and applicable, practical change strategies tailored to relevant barriers and facilitators [23–25]. To facilitate this process in primary dental care, National Health Service (NHS) Education for Scotland established the Translation Research in a Dental Setting (TRiaDS) initiative in 2008 [26]. The multi-disciplinary TRiaDS collaboration has developed a theoretically driven framework to integrate production, dissemination, and evaluation of SDCEP guidance. The TRiaDS framework provides a systematic approach, underpinned by theories of behaviour change, to identify gaps in practice, develop translational interventions, and test intervention strategies in primary care practice [26]. This paper describes a mixed-methods study design using the TRiaDS approach to investigate barriers and facilitators to implementation of guidance-recommended care for the prevention and management of caries (PMC) in primary dental care in Scotland.

The Behaviour Change Wheel was selected as the overarching theoretical framework for this study given the wheel's direct path from behavioural assessment to selection and development of theory-based intervention strategies [27, 28] and the clinician-led, highly autonomous environment of dental practice. Consistent with usual operationalization processes of the wheel, we applied the Theoretical Domains Framework (TDF) [29–31] to identify specific domains (e.g. knowledge, skills, environmental context and resources) influencing target behaviours. Identified domains were integrated into the wheel through the capability, opportunity, motivation-behaviour (COM-B) model [27, 28]. The COM-B enabled structuring of policy-level barriers and facilitators to target behaviours and identification of change strategies most likely to improve practice (e.g. audit and feedback) by influencing the TDF domains salient to target behaviours. A detailed discussion of this process as applied to caries prevention and management follows in the “Methods” section.

## Methods

**Fig. 1:**
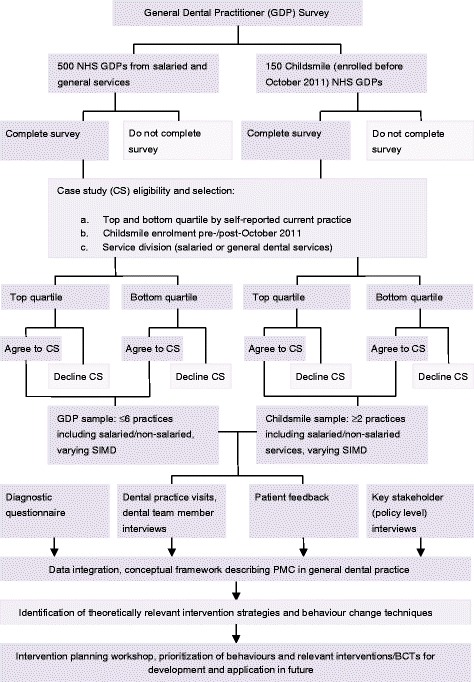
Study design

This study used a convergent mixed-methods design [32–34] incorporating the TRiaDS approach [26] and Behaviour Change Wheel. Methods (Fig. 1) comprised a diagnostic questionnaire, in-depth practice case studies, patient interviews, and system-level informant interviews. Findings were integrated into a single matrix describing factors influencing PMC practice and used with key stakeholders to collaboratively identify theoretically relevant interventions to support further implementation of recommended PMC practice.

### Identifying target behaviours

**Fig. 2:**
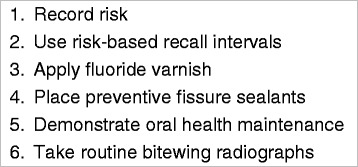
Prioritized PMC behaviours

Caries prevention and management encompasses a range of behaviours, and prioritization was necessary to target our assessment. Behaviour Change Wheel and TDF protocols recommend balancing the number of behaviours assessed to improve targeting and specificity of interventions and so their likelihood of success [28]. A prioritization exercise to identify key PMC behaviours was conducted with the TRiaDS methodology group comprising practitioners, policy makers, implementation scientists, programme leaders, and experts in dental public health. Group members prioritized PMC behaviours for children and adults according to current compliance, anticipated ability to increase the behaviour, measurability, and public health gains. Six behaviours (Fig. 2) were selected as key best-practice recommendations. Recording risk, risk-based recall intervals, and taking bitewing radiographs were all included as essential components of basic risk assessment. Demonstration (as opposed to discussion) of oral health maintenance was included as a universally recommended, but little practised, component of routine preventive care. Fluoride vanish application and preventive fissure sealant placement ranked highly on all criteria and scored the highest on public health gains, particularly given the ongoing investment in Childsmile as a public health programme.

### Sample

#### Diagnostic questionnaire

The initial study sample included 651 dentists in general dental practice providing care through NHS Scotland; 150 of whom participated in Childsmile prior to October 2011 when a fee for fluoride varnish was introduced and Childsmile became a universal programme. Dentists providing Childsmile before October 2011 were oversampled to enable comparison of recommended PMC by early participation in the initial phase of the programme. Using computer-generated random numbers at the practice and dentist level, general dental practitioners (*N* = 501) were selected from a publicly available list published by Practitioner Services Division of NHS National Services for Scotland [35]; Childsmile dentists (*N* = 150) were selected from Information Services Division of NHS National Services for Scotland database of Childsmile demonstration practices. Sample size was calculated based on a standard response rate of 50 % as in previous questionnaires conducted by PMC study team members [21, 22, 36, 37]. Sample size was calculated using the recognized sample size method of 10 data items per predictor for a regression model with up to 20 predictors [38].

#### Case studies

A purposive, theoretical sample of case study practices (*N* = 8) was identified from questionnaire respondents. Case study practices were selected from the highest and lowest performing quartiles of self-reported guidance-recommended practice. Selection criteria also included Childsmile enrolment, service division (salaried/general practice), and Scottish Index of Multiple Deprivation (SIMD) quintile ranking as characteristics known to influence caries burden and preventive care delivery [11, 12, 19, 22, 39]. Invitations were sent to eligible practices until four high- and four low-performing practices were recruited. Patient feedback was initially conducted in the first four recruited practices (two high, two low) but later extended to all practices.

#### System-level informant interviews

System-level informants (*N* = 4) were identified by the PMC study team and TRiaDS methodology group for their expertise, involvement, or leadership in PMC and preventive oral health. Informants represented patient, practice, and policy interests at the system (service development, planning, delivery, regulation) level. The sample was small as informants were being asked to provide general PMC feedback, not detailed, behaviour-specific assessment as in the diagnostic questionnaire or case studies.

### Consent and ethical review

Ethical review and approval for the PMC study was provided by East of Scotland Research Ethics Committee 1. Research and development management and approval was conducted through NHS Research Scotland Permission Coordinating Centre.

### Procedure

#### Diagnostic questionnaire

Current practice of each behaviour was measured as yes/no responses to “During a routine visit I am likely to:” across primary (i.e. deciduous), mixed, and adult dentition types. The timing of routine visits was not specified as intervals should be risk-based and therefore variable [16, 17].

Beliefs were assessed using the then available 12 domain TDF [29]. To produce a focussed, concise questionnaire, *nature of the behaviour* was eliminated a priori consistent with other quantitative applications of the TDF [40, 41]. Domains were further reduced in a second prioritization exercise with the TRiaDS methodology group by considering their relevance to each target behaviour. Domains *not* reasonably expected to be associated with each behaviour were eliminated. For universal behaviours (recording risk, risk-based recall, demonstration of oral health maintenance), all prioritized domains (Additional file 1) were included in the questionnaire; domains prioritized specific to children (e.g. cooperation as social influence) were only assessed for child-focused behaviours (fluoride varnish, preventive fissure sealants). Bitewing radiographs were assessed by dentition type as the behaviour is recommended for all patients over 4 years old, but barriers were expected to differ between children and adults. Domains were assessed on 5-point Likert scales using 1–5 established items from existing literature [29]. Specialists reviewed items to ensure clarity of the behaviour and item fit within domains. As the number of items remained high, each participant was only assessed on two behaviours. Three versions of the belief assessment were produced: recording risk and applying fluoride varnish, risk-based recall and placing preventive fissure sealants, and demonstrating oral health maintenance and taking bitewing radiographs. Similar behaviours were separated to reduce bias.

Practice characteristics included demographic information, practice structure and staff roles, Childsmile enrolment, quality assurance systems, and perceived relevance of PMC guidance. Items were selected using the Consolidated Framework for Implementation Research (CFIR) [42] as a complement to the TDF to increase specificity of organizational assessment [43]. Dentist characteristics were assessed using standard TRiaDS protocol [21, 36].

The questionnaire (Additional file 2), including the three versions of belief assessment, was reviewed for comprehension and face validity with a convenience sample (*N* = 5) of dental professionals and oral health researchers.

The questionnaire was administered by post with a reminder and questionnaire sent at 2 weeks, a reminder phone call with opt-out option at 4 weeks, and a final reminder and questionnaire at 6 weeks. No incentive was offered for completion.

#### Case studies

Data collection tools (Additional file 3) were developed by the five-member qualitative workgroup, a sub-group of the PMC study team comprising social sciences, health services, and health psychology researchers. Patient visit observations, interviews, and focus groups were audio recorded and transcribed. Practice observations were recorded as written field notes. Researchers conducting the case studies were blinded to practice performance (high/low) of guidance-recommended care.

Practice visits and non-participant observation of three routine adult exams were conducted in each case study to assess context of care. As observation was used to produce a general description of preventive care (rather than care specific to each behaviour), only adult exams were observed. A structured observation guide based on the TDF and CFIR was developed.

Semi-structured interviews with respondent dentists (*N* = 8) and up to three other dental team members (e.g. dental nurses, practice managers, vocational trainees, receptionist) (*N* = 23) assessed guidance use and barriers and facilitators to recommended PMC. Questions were not domain specific in order to allow domains excluded from the questionnaire to emerge and to elicit more contextual detail. Interview schedules were mapped to the TDF to ensure coverage of all domains. The interview schedule was reviewed for comprehension and face validity by the same convenience sample (*N* = 5) as the questionnaire.

Patient feedback was planned through a series of focus groups embedded in four case study practices. The focus group schedule emphasized patient experiences of PMC (nature of the behaviour), barriers and facilitator to PMC (beliefs about consequences), and patient expectations (social influence). As focus group attendance was low, all practices were invited to provide patient feedback through structured telephone interviews. Interviews were based on the focus group schedule, emphasizing the same domains but using a less open-ended approach to reduce time required for participation. Patient representatives from the study management team provided in-depth feedback and guidance in recruitment and conduct of patient focus groups and interviews. Patients were provided a £10 voucher in appreciation of their participation.

#### System-level informant interviews

Semi-structured interviews were conducted with key informants in policy, service delivery, and service development. Informants were identified by the study team based on their expertise and involvement in oral health policy and delivery systems. Interview schedules were developed using the COM-B and intervention and policy categories in the Behaviour Change Wheel [28]. Interviews were conducted by telephone, audio recorded, and transcribed.

### Analyses

#### Diagnostic questionnaire

Reliability analyses (Cronbach’s alpha) of TDF domains assessed for each behaviour were conducted. In domain items with alpha >0.60, items were combined into single scales. Responses were analysed using simple descriptive statistics, univariate analyses, and logistic regression in SPSS 22. Comparisons and regressions were conducted for primary, mixed, and adult dentition types based on self-reported current practice. For each of the six behaviours, TDF domain scales and demographic data (individual and practice level) were compared by self-reported provision of guidance-recommended care. Significant (*p* ≤ 0.05) variables from univariate analyses were entered into a logistic regression by best/not best practice and dentition type. A forward stepwise (Likelihood ratio) method was used to manage co-linearity issues with TDF scales as sample sizes were restricted to respondents in each questionnaire version.

#### Case studies

Observational data were collated in Excel. Field notes were supplemented with visit transcriptions. Practice characteristics and observed care, including factors influencing best practice, were analysed thematically across cases.

Dental team interviews were analysed using a TDF-based coding guide (Additional file 4). Responses were coded by behaviour, associated domains, and function as a barrier or facilitator to recommended care. Emergent themes were identified and included in the coding guide as they developed. Twenty percent of interviews were double coded to assess coder reliability and consistency. Individual analyses were produced for each case study practice including detailed description of current behaviour, pertinent quotes, and framework matrices of codings for the six behaviours and emergent themes. Findings across cases were summarized to describe beliefs influencing each behaviour. Cross case summaries included frequency of beliefs, presence of conflicting beliefs, and participant expressions of the strength of their beliefs as an impact on behaviour [44–46].

Patient feedback was analysed using a TDF-based coding guide (Additional file 4) with emergent themes specific to patient responses. Twenty percent of patient feedback was double coded for reliability and consistency. Due to uneven spread of feedback across practices (range of 0–8 patients per practice), patient feedback was aggregated across all cases. Cross case findings from focus groups and interviews were compiled in a single summary of patient characteristics, current behaviour, experiences related to PMC, pertinent quotes, and emergent themes.

Dental team and patient data were managed and analysed in Nvivo 10.

#### System-level informant interviews

Informant interviews were coded by emergent themes (e.g. capacity, data systems) as barriers or facilitators to recommended care. Responses were coded into themes by policy, system, and intervention characteristics in the Behaviour Change Wheel and then mapped to the COM-B to enable later integration with questionnaire and case study data. Data were managed in Excel.

### Data integration

A day-long workshop with study team members was held to integrate findings from individual study components, identify associated domains (i.e. any domain assessed as influencing behaviour), and appraise salient domains (i.e. domains with the greatest influence on behaviour or potential for intervention). Study team members familiarized themselves with findings using questionnaire, case study, patient feedback, and system-level informant interview summary reports. Reports included methods, in-depth analyses, summary findings of behaviour-specific beliefs, associated domains, and domain function as a barrier or facilitator. The workshop followed a three-step process. First, associated domains from each study component were reviewed individually for clarification, discussion of unexpected findings, and consideration of differences between study components. Second, associated domains were entered into a matrix describing barriers and facilitators across study components for the six key behaviours. Based on emergent themes around information exchange and advice from the case studies (general preventive care) and patient feedback (oral health advice, oral health maintenance), *oral health advice* was added as an additional behaviour to the matrix. Third, associated domains in the matrix were prioritized into salient domains based on critical appraisal of frequency within and across study components, degree to which respondents and components agreed or disagreed about the domain, and evidence of strong beliefs impacting the behaviour [44–46].

Salient domains in the finalized matrix were mapped to COM-B categories, theoretically relevant intervention strategies in the Behaviour Change Wheel, and associated behaviour change techniques. Behaviour change techniques were defined using the Behaviour Change Technique Taxonomy, version 1 [28, 47].

### Intervention planning

Key stakeholders met in a half-day workshop to review findings, intervention strategies, and behaviour change techniques and prioritize approaches to improve preventive care delivery in primary dental care. Stakeholders (*N* = 9) comprised patient-, practice-, and policy-level representatives. Stakeholders familiarized themselves with study findings through brief behavioural summaries (including pertinent quotes and routine data where available) and the barrier/facilitator matrix. Intervention planning followed a three-step nominal group technique [48]. First, stakeholders reviewed the findings for each behaviour and discussed differences in priorities by stakeholder group, current compliance with guidance recommendations, anticipated ability to increase the behaviour, and anticipated public health gains. Second, behaviours were prioritized using established criteria of appropriateness, relevance, feasibility, and potential impact [49–51]. Behaviours to target with interventions were agreed by consensus. Third, based on prioritized behaviours, stakeholders reviewed theoretically relevant intervention strategies and behaviour change techniques. Strategies and techniques were appraised using the affordable, practical, effective, acceptable, safe, equitable (APEASE) criteria [28] in order to develop consensus on approaches most likely to support further PMC guidance implementation of PMC.

## Results

### Participants

#### Diagnostic questionnaire

Of the original 651 dentists invited to participate, 73 were ineligible (left practice, retired, deceased, long-term leave), 59 declined taking part, and 323 questionnaires were not returned. A total of 196 dentists responded (34 % response rate). Response rates did not differ between the three belief assessment sections. Responders and non-responders did not differ by health board, SIMD, or Childsmile enrolment before October 2011.

Individual respondent characteristics are summarized in Table 1. On average, respondent practices had a 78 % of patients registered on NHS, 20 % of patients under 16 years of age, and three dentists working in the practice. Under half of practices employed a hygienist (44.4 %), therapist (35.7 %), or extended-duty dental nurse (46.4 %). Two thirds of practices (66.3 %) had a practice manager. Most (79.6 %) practices self-reported as Childsmile practices with 39.6 % delivering Childsmile before October 2011. Just over a third of practices had a patient feedback system (38.1 %) or undertook routine quality assurance (39.2 %).

**Table 1:** Diagnostic questionnaire respondent characteristics

	Value	Number (*n*)
Gender		196
Male	56.1 %	(110)
Female	43.9 %	(86)
Age (years)		194
Median	37.5	
Mean (SD)	40.0 (10.9)	
Range [Q1, Q3]	25–65 [30, 49]	
Number of years qualified		193
Median	14	
Mean (SD)	16.47 (11)	
Range [Q1, Q3]	0–42 [7, 25]	
Role		191
Principal	37.7 %	(72)
Associate	57.1 %	(109)
Salaried	4.2 %	(8)
Other	1 %	(2)
Only practice where work		196
Yes	85.7 %	(168)
No	14.3 %	(28)
Primary practice setting		186
General dental service	86 %	(160)
Community service	0 %	(0)
Salaried service	3.8 %	(7)
Private	4.2 %	(8)
Other	0.5 %	(1)
Sessions (0.5 day) worked per week		192
Median	9	
Mean (SD)	8.4 (2.02)	
Range [Q1, Q3]	0.4–14 [8, 10]	
Patient list size		179
Median	2000	
Mean (SD)	2532 (3240)	
Range [Q1, Q3]	20–30,000 [1400, 3000]	

#### Case studies

Case study practices were selected by best practice (highest quartile for guidance-recommended care delivery) and not best practice (lowest quartile). Two practices were Childsmile practices before October 2011; one practice did not identify as a Childsmile practice. All practices were in the General Dental Service with at least 60 % of patients seen on NHS. Practices spanned all SIMD quintiles; five were SIMD 3 or below. Twenty-nine team members were interviewed across the eight practices. In each case, interviewees included the dentist who completed the questionnaire and one dental nurse. Other team members interviewed included additional dentists and dental nurses, three practice managers, two extended-duty nurses, two vocational trainees, one receptionist, and one hygienist.

Of the 19 patients who took part, most (73.7 %) were female, 30–50 years old (52.6 %). Just under half (47.4 %) had children under the age of 16. Patients attended seven of the eight case study practices.

#### System-level informant interviews

Key informants (*N* = 4) included representation from the Scottish Government, NHS Education for Scotland, British Dental Association, dental public health, Childsmile, guidance development, general and salaried dental services, patient representatives, and general dental practitioners.

#### Intervention planning workshop

Intervention planning was conducted by nine key stakeholders and six study team members including the workshop facilitator. Key stakeholders comprised guidance development, continuing dental education, the Scottish Government, Childsmile, dental public health, dental practice regulation, dental practitioners, and lay representatives. Study team members attending all participated in the previous integration workshop.

### Diagnostic questionnaire

Current practice, as the percentage of respondents who “during a routine dental visit” were “likely to” deliver the six behaviours, is summarized in Table 2. Self-reported practice tended to align with guidance-recommended care.

**Table 2:** Self-reported PMC in routine general dental practice by dentition type

	Primary dentition (%)	Mixed dentition (%)	Adult dentition (%)
Record risk	82.1	84.2	48.2
Risk-based recall intervals	71.4	73.2	62.2
Apply fluoride varnish	80.9	67.4	17.9^a^
Place fissure sealants	16.7^a^	87.0	22.5^a^
Demonstrate OH maintenance	86.8	90.5	70.5
Take bitewing radiographs	9.4	63.3	93.3

^a^No agreed guidance recommendations about best practice

Differences on TDF scales between best-practice and not best-practice dentists were examined using *t* tests. Logistic regression split by dentition type identified predictive domains for recording risk, risk-based recall, applying fluoride varnish, demonstrating oral health maintenance, and taking bitewing radiographs (Additional file 5). The *behavioural regulation* domain was the most consistent predictor of performance, but the study team agreed at analysis not to treat it as associated due to item similarity with behavioural measures. Although variables identified in univariate and logistic analyses differed, given the general diagnostic nature of the questionnaire, any domain by best practice/not best practice was carried forward for consideration at the integration.

### Case studies

#### Practice observations

Twenty-four adult patient visits were observed across the eight case study practices. Observed preventive behaviours included recording risk, risk-based recall, and bitewing radiographs. All visits included a dental nurse supporting the dentist with record keeping. Practices displayed standard NHS information, practice-specific patient leaflets, and appointment policies. Display of oral health information or promotion of oral health initiatives like Childsmile was extremely rare. In all but two practices, the majority of information displayed was to promote cosmetic or private pay procedures.

#### Dental team member interviews

Across cases, all team members thought preventive care is important, benefits patient oral health, and is part of every team member’s role. Within individual cases, dental teams reflected the dentist’s beliefs about barriers and facilitators to PMC behaviours (e.g. scepticism regarding efficacy of fissure sealants). Team members’ beliefs were more divergent on the emergent theme of *general preventive care*. Dental nurses, and extended-duty dental nurses in particular, emphasized benefits of prevention (beliefs about consequences), prevention as integral but underutilized skill set in their role (social and professional role and identity), and a desire to provide more preventive care. Respondents had positive attitudes toward guidance but emphasized guidance as often too long, complicated, and not universally applicable or practical.

Barriers and facilitators to PMC behaviours and general preventive care differed little between high- and low-performing practices. Low-performing practices tended to emphasize barriers such as patient expectations (social influence), lack of time (environmental context and resources), and few perceived benefits (beliefs about consequences) and did not identify preventive oral health as strongly within their roles (social professional role and identity). The same barriers were identified to a lesser extent by high-performing practices. Few domains acted exclusively as a facilitator or barrier to PMC behaviours. Most domains (e.g. social influence) functioned simultaneously as barriers (e.g. parents refuse fluoride varnish) and facilitators (parents request fluoride varnish) to PMC behaviours. Duality applied across dentition types, even when some feature of the domain (e.g. a child versus an adult patient) changed. Given the consistency of domains influencing behaviour in both high- and low-performing practices and often dual function of those domains as barriers and facilitators, associated domains were described across all cases rather than by best practice/not best practice. Domains associated with PMC behaviours and general preventive care are summarized in Additional file 5.

#### Patient feedback

Patients identified the most important factors for preventive oral health as tooth brushing and cleaning for adults and supervising tooth brushing and limiting sugar intake for children. The discussion of barriers and facilitators to PMC was framed by patient’s experiences, including self-care (oral health maintenance, diet, assistance/supervision of children), advice and information from their dental team, and other sources of information (e.g. media). For both adults and children, patients identified multiple long-term benefits of preventive oral health care but were unsure about the efficacy of their self-care techniques, were anxious about dental appointments, and struggled with care (particularly brushing) of children’s teeth. As few patients had experienced hands-on demonstrations of oral health maintenance, few expected this type of instruction but nearly all of them anticipated it as highly beneficial and were eager to receive such care. Patient experiences and expectations centred primarily on informational exchange and were categorized in emergent *oral health advice* and *oral health management* themes. Associated domains are summarized in Additional file 5.

### System-level informant interviews

System-level informants emphasized improvements in preventive oral health made in recent years and were optimistic about narrowing the gap between patients in high- and low-deprivation areas. Persistent concern about caries, PMC, and disproportionate burden of disease among higher deprivation populations was common. Gains in PMC were attributed to community-based preventive care programmes (particularly in primary schools) and increased delivery of oral health advice in dental practices. Eighteen themes emerged as barriers, facilitators, or both (Additional file 5); themes related to all three behavioural determinants in the COM-B model.

### Data integration

#### Step 1

After initial review of associated domains from each component, attendees made two consensus recommendations. First, that system-level informant data be considered separately from questionnaire and case study findings as it was more generalized and policy focused. Second, that case study findings for dental team and patient feedback be considered separately given the differences in practice and patient perspectives.

#### Step 2

**Fig. 3:**
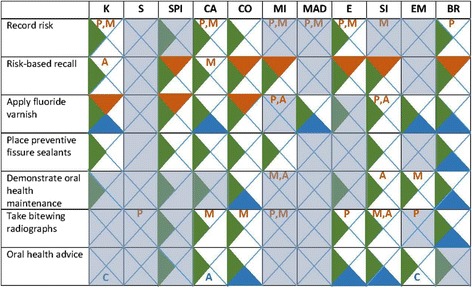
Associated domains influencing PMC practice. Legend: *orange* questionnaire, *green* case studies, *blue* patient feedback, *K* knowledge, *SPI* social professional role and identity, *CA* beliefs about capabilities, *CO* beliefs about consequences, *MI* motivation, goals, and intent, *MAD* memory, attention, and decision making, *E* environmental context and resources, *SI* social influence, *EM* emotion, *BR* behavioural regulation, *P* primary dentition, *M* mixed dentition, *A* adult dentition or adults, *C* children

Associated domains from the questionnaire, dental team member interviews, and patient feedback were entered into a matrix comprising the six study behaviours and *oral health advice* theme (Fig. 3). Domains without convergence in two or more components (e.g. beliefs about consequences only identified in dental team member interviews for recording risk) were eliminated. In the case of fissure sealants where convergence only occurred within *behavioural regulation*, all domains were carried forward for further consideration.

#### Step 3

**Fig. 4:**
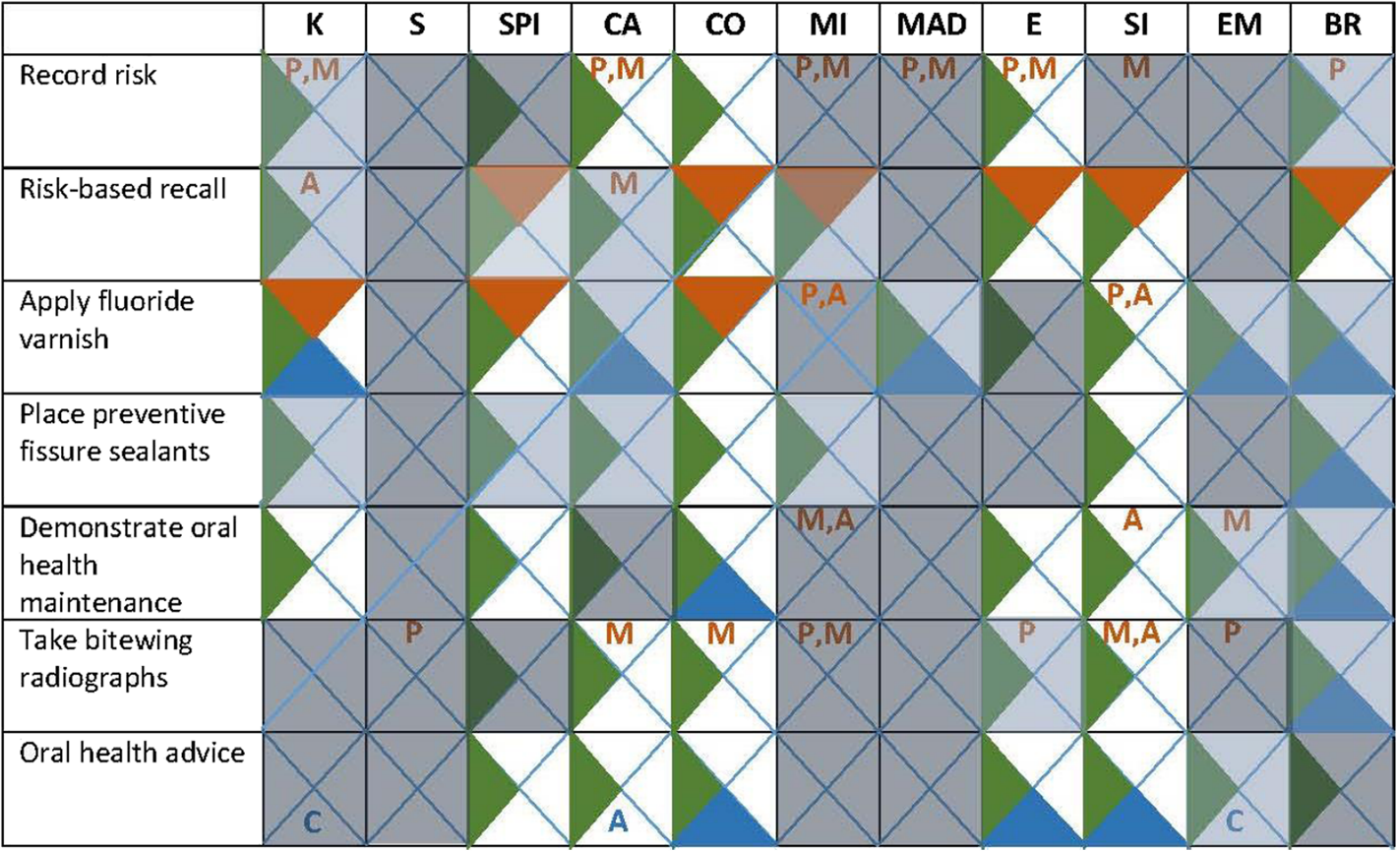
Salient domains influencing PMC practice. Legend: *orange* questionnaire, *green* case studies, *blue* patient feedback, *K* knowledge, *SPI* social professional role and identity, *CA* beliefs about capabilities, *CO* beliefs about consequences, *MI* motivation, goals, and intent, *MAD* memory, attention, and decision-making, *E* environmental context and resources, *SI* social influence, *EM* emotion, *BR* behavioural regulation, *P* primary dentition, *M* mixed dentition, *A* adult dentition or adults, *C* children

Associated domains were appraised into salient domains (Fig. 4) based on frequency of beliefs, presence of conflicting beliefs, and the strength of beliefs’ impact on behaviours [44–46]. Salient domains were agreed by consensus. Further 18 cells were removed, primarily as respondent beliefs (i.e. the domains) were mentioned frequently but without evidence of strong influence on PMC behaviours. Three cells eliminated in step 2 (demonstration of oral health maintenance—social professional role and identity, environmental resources and context; oral health advice—social professional role and identity) were reinstated as salient domains given the strength and frequency of dental team member feedback.

Salient domains were annotated according to behaviour, function as barrier or facilitator, and study components (Additional file 6). The final matrix was circulated to attendees to confirm content and constituted this study’s summative findings of barriers and facilitators to PMC in general dental practice.

#### Mapping to intervention strategies and behaviour change techniques

**Fig. 5:**
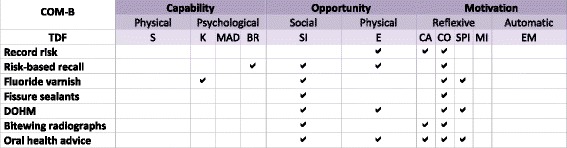
Salient domains from the TDF mapped to the COM-B model

**Fig. 6:**
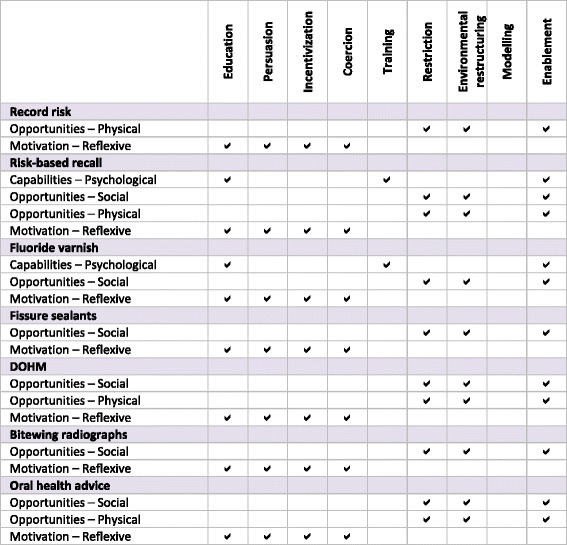
Barriers and facilitators from the COM-B mapped to intervention strategies in the Behaviour Change Wheel

Domains from the conceptual framework were mapped through the COM-B (Fig. 5) to intervention strategies in the Behaviour Change Wheel (Fig. 6). As the COM-B consolidated domains into broader categories of *capability*, *opportunity*, and *motivation*, most intervention strategies (except training and modelling) were common across behaviours. Available behaviour change techniques were identified based on theoretically relevant intervention strategies, available evidence summarizing effective behaviour change techniques [28], the Behaviour Change Technique Taxonomy [28, 47], and expert recommendations from implementation scientists on the TRiaDS group. Specific techniques included feedback on behaviour; feedback on the outcomes of the behaviour; self-monitoring of behaviour; educational interventions based on behaviour modelling and credible sources; action planning; provision of scripts to model, prompt, and reinforce behaviour; and goal setting (in addition to feedback) to help practitioners gauge actual as to perceived performance.

### Intervention planning workshop

Stakeholders reviewed the study matrix and resolved that while guidance recommendations should be based on dentition type, interventions should be developed by patient age group given current service delivery and remuneration structures. Using these parameters, stakeholders prioritized the 6–12-year-old age group (given the transition from primary to adult dentition, updated guidance recommends universal fissure sealant placement, reduced access to community-based oral health programmes in this age group) for initial development of interventions to support improved PMC practice. Stakeholders next prioritized study behaviours for 6–12-year-olds using established criteria of appropriateness, relevance, feasibility, and potential impact [49–51]. Criteria were assessed from all stakeholder perspectives. Fissure sealant ranked highest and was agreed as the intervention target. Discussion frequently included fluoride varnish, and while preventive fissure sealant placement was the clear priority for 6–12-year-olds, stakeholders were in agreement that where suitable, interventions could target both behaviours or be adapted from one to the other.

In the final stage of the workshop, theoretically relevant intervention strategies to improve fissure sealant placement were discussed. Two interventions were recommended for future implementation: audit and feedback at the provider level (persuasion, incentivization, coercion, training, enablement) and a preventive oral health awareness campaign at the patient level (enablement). In APEASE appraisal, equity did not score well as interventions were practice based and patients who rarely or never attend the dentist would not benefit. However, given other criteria and continuing increases in registration rates, stakeholders were unanimous that both interventions be developed for implementation. Behaviour change techniques agreed for the audit and feedback intervention were action planning, feedback on behaviour and outcomes, goal setting, problem solving, review of goals and outcomes, and self-monitoring. The primary technique in the patient intervention would be social support to encourage patients (parents) to discuss fissure sealants and fluoride varnish with dental care providers and enable patient requests for preventive care or additional oral health advice.

## Discussion

### Study strengths

The mixed-methods approach identified well-defined barriers and facilitators to preventive care; consistent application of the theoretical framework through each component to data integration and intervention planning resulted in specific, targeted interventions suitable for implementation.

The questionnaire quantified beliefs of dentists following best practice versus not best practice and identified high- and low-performing practices for case study selection. At the time of writing the questionnaire, the TDF had only been applied in two quantitative studies [40, 41] and demonstrated varying degrees of internal consistency in domains and either one [41] (propensity to act) or three [40] (capability, opportunity, motivation) factor explanatory models. Subsequent quantitative applications [22, 52, 53] have shown good discriminant validity, collated a wider array of domain items, and demonstrated levels of internal consistency similar to our findings.

Consistent with case study methodology [54, 55], case studies provided detailed context and nuance to decisions and practices around PMC care delivery and produced information essential to appraisal and identification of salient domains identified in the questionnaire. For example, the case studies emphasized the duality of domains as barriers and facilitators in both high- and low-performing practices and brought different domains (particularly social influence, professional role, and identity) to the forefront than were anticipated from previous research (remuneration within environmental context and resources, motivation and intent, beliefs about consequences).

Inclusion of patient feedback emphasized differences between patient and dentist expectations of preventive care and identified patients’ social influence as an intervention approach to improving practitioner behaviour. This finding in particular highlights the potential benefits of developing interventions to encourage and empower patients to influence the delivery and content of preventive care.

System-level informant interviews provided a broad policy context which helped identify salient domains and integrate intervention planning with ongoing initiatives including PMC guidance, audit, and service development. While the number of interviews was limited, interventions in all three categories of the COM-B were clearly identified. Improvement strategies emerging from the interviews (particularly audit) were consistent with those discussed in the intervention planning workshop.

Application of the Behaviour Change Wheel as our single theoretical framework provided a common language across study component findings and a logical structure for data integration and intervention planning. Component findings converged clearly, and qualitative findings emphasized and explain barriers and facilitators within each domain. Using the wheel, salient domains were easily mapped to appropriate, evidence-based intervention techniques framed in policy terms familiar to stakeholders. The practical, direct path from barriers and facilitators to interventions greatly helped to parse the diverse data collected and identify specific techniques to improve care.

### Study limitations

While six target behaviours were prioritized from recommended practices, determinants varied by patient dentition types (primary, mixed, adult). This complexity necessitated design adaptations including belief assessment of only two behaviours per questionnaire respondent and discussion of preventive care in general, using the six behaviours as specific examples or instances, in the case studies. These were practical compromises for initial identification of barrier and facilitator PMC in general dental practice; in the future, we would recommend focusing on a narrower range of behaviours or a single patient age group to improve comparability of findings

The response rate in our questionnaire (34 %) was lower than the anticipated 50 %. Efforts to improve the response rate (telephone call reminder, additional posted copy of questionnaire) helped produce a further 69 responses after the second reminder but did not achieve the desired rate. Responses were sufficient for statistical analyses and varied enough to identify case study practices but make the sample more vulnerable to self-selection bias.

Assessing PMC practice by self-report revealed higher than expected rates of recommended care delivery given nationally reported rates of fluoride varnish application to 23 % of 2–5-year-olds [56] and preventive fissure sealant placement to 29 % of primary 7 students (mean age 11.5 years) [57]. Due to data availability, direct comparison of self-reported with routine data for respondent dentists was not possible prior to analyses and integration. Routine data did reveal higher rates of fluoride varnish among our sample (50.7 % overall, 56.3 % among high performers, 21.5 % among low performers) but similar fissure sealant rates (20 % overall, 27.1 % among high performers, 13.8 % among low performers) to national averages. Although routine data demonstrated lower rates of recommended care, the differences between high and lower performers by self-report were sufficient to enable meaningful comparison. Discrepancies between self-report and routine data may have been due to assessment of behaviours at “a routine visit” rather than fixed intervals, assessment by dentition types rather than age ranges, or a respondent group who provided more recommended care and self-selected to complete the questionnaire. Anecdotal evidence from system-level informants and key stakeholders indicated that some dentists are providing fluoride varnish and fissure sealants but not claiming for these treatments due to complexities in the payment system including documentation and age-based exclusions. These reports could not be validated in this study but were consistent with routine data findings for fluoride varnish and came up frequently enough to encourage further investigation.

The Behaviour Change Wheel helped structure our study but presented some challenges and limitations in application. First, operationalizing the framework within a multi-disciplinary study team, particularly familiarization with the TDF and COM-B model, took concentrated effort and structured communication among team members. Discriminating between TDF domains necessitated the development of clear guidelines for team members conducting analysis and interpretation of data. Second, once salient domains were mapped to relevant intervention strategies, many of the interventions and behaviour change techniques overlapped raising the question whether detailed assessment of TDF or even COM-B categories was necessary. Reflecting upon this with the TRiaDS group, the study team generally felt that while the interventions were not discrete to individual behaviours, the theoretical grounding of interventions and potential to adapt interventions to multiple behaviours outweighed crossover of intervention strategies within the framework. Third, organizational characteristics influencing PMC practice did not emerge as strongly as expected. While efforts to improve organizational assessment through the CFIR were made, the depth in which organizational characteristics were evaluated was practically limited by the number of behavioural domains assessed. Mapping the CFIR to the TDF helped remediate this issue and identified several specific characteristics (e.g. other dentists performing the behaviour, established feedback and quality assurance systems) common to practices delivering recommended care. Focus on fewer behaviours may have enabled a more detailed assessment of organizational climate and culture and identification of practice-level influences on PMC. However, practical compromises were necessary to provide sufficient breadth in this initial assessment of PMC and it is possible that organizational factors were not as influential as expected.

## Conclusions

Key stakeholders and study team members coproduced specific, theoretically based, testable interventions representative of interests within and across patient, practice, and policy groups. Interventions have been favourably appraised by all stakeholders as implementation ready, complementary to ongoing national guidance and professional development initiatives, and likely to improve translation of recommended care into routine primary practice—reducing both the incidence and prevalence of caries in children and adults. Across mixed-methods components, our findings emphasize the duality of domains as barriers and facilitators, the strength of patient influence on practitioner behaviour, and the benefit of integrating multiple levels of interest when planning interventions in a dynamic yet resource-constrained preventive care delivery system. The development of simultaneous patient- and practice-focussed implementation strategies to improve preventive services in primary care is highly relevant to translational research across health services.

Findings are actively being used to review, update, and develop implementation tools accompanying the revised Scottish Dental Clinical Effectiveness guidance on child caries to be published in 2016. In collaboration with NHS Education for Scotland and Childsmile, TRiaDS has developed and piloted pre-approved national audits for fluoride varnish and fissure sealants which will be available with publication of the SDCEP guidance. Future plans include testing the use of audit and feedback interventions for preventive care (fluoride varnish, fissure sealants, bitewing radiographs) and a practice-based campaign to promote patient awareness, support, and requests for PMC.

## Additional files

Additional file 1: **Prioritized domains included in diagnostic questionnaire.** TDF domains assessed for target behaviours.

Additional file 2: **Diagnostic questionnaire.** Assessment of current practice, beliefs, and demographic information.

Additional file 3: **Case study and patient feedback data collection tools.** Qualitative data collection tools.

Additional file 4: **Dental team member interview and patient feedback coding guides.** Qualitative TDF coding guides.

Additional file 5: **Associated TDF domains identified in individual study components.**

Additional file 6: **Salient domains matrix.** Annotated domains influencing targeted PMC behaviours.

## Abbreviations

APEASE: affordable, practical, effective, acceptable, safe, equitable
CFIR: Consolidated Framework for Implementation Research
COM-B: capability, opportunity, motivation-behaviour
NHS: National Health Service
PMC: prevention and management of caries
SDCEP: Scottish Dental Clinical Effectiveness Programme
SIGN: Scottish Intercollegiate Guidelines Network
SIMD: Scottish Index of Multiple Deprivation
TDF: Theoretical Domains Framework
TRiaDS: Translation Research in a Dental Setting
