# Supplementary material for: Patient-, organization-, and system-level barriers and facilitators to preventive oral health care: a convergent mixed-methods study in primary dental care
Source: Implement Sci. 2016 Jan 12;11:5. doi: 10.1186/s13012-015-0366-2 (PMC4710040; doi:10.1186/s13012-015-0366-2)
Supplement: Supplementary file 1 — Prioritized domains included in diagnostic questionnaire. TDF domains assessed for target behaviours. [file 13012_2015_366_MOESM1_ESM.pdf]

Additional file 1
Prioritized domains included in diagnostic questionnaire

TDF domains for each of the six study target behaviours, prioritized for inclusion in questionnaire

	K	S	SPI	CA	CO	MI	MAD	E	SI	EM	BR
**Record risk**	✔		✔	✔	✔	✔	✔	✔	✔		✔
**Risk-based recall intervals**	✔	✔	✔	✔	✔	✔	✔	✔	✔		✔
**Apply fluoride varnish**	✔		✔		✔	✔		✔	✔		
**Place fissure sealants**	✔	✔	✔	✔	✔	✔		✔	✔		
**Demonstrate OH maintenance**	✔		✔	✔	✔	✔		✔	✔	✔	
**Take bitewing radiographs**	✔	✔	✔	✔	✔	✔		✔	✔	✔	

K – knowledge; SPI – social professional role and identity; CA – beliefs about capabilities; CO – beliefs about consequences; MI – motivation, goals, and intent; MAD – memory, attention, and decision making; E – environmental context and resources; SI – social influence; EM – emotion; BR – behavioural regulation
