# Supplementary material for: Patient-, organization-, and system-level barriers and facilitators to preventive oral health care: a convergent mixed-methods study in primary dental care
Source: Implement Sci. 2016 Jan 12;11:5. doi: 10.1186/s13012-015-0366-2 (PMC4710040; doi:10.1186/s13012-015-0366-2)
Supplement: Supplementary file 2 — Diagnostic questionnaire. Assessment of current practice, beliefs, and demographic information. [file 13012_2015_366_MOESM2_ESM.pdf]

Additional file 2
Diagnostic questionnaire

Prevention and management of caries in general dental practice

GDP Questionnaire

September – October 2012

This study is funded by the Chief Scientist Office, Health Care Improvement Grant, Ref. CZH/3/27

Section 1: Your current practice

Q1 During a routine dental visit for the following patients, I am likely to:

	Child with deciduous teeth		Child with permanent and deciduous teeth		An adult	
	Yes	No	Yes	No	Yes	No
a. Record caries risk	☐	☐	☐	☐	☐	☐
b. Choose a recall interval based on caries risk	☐	☐	☐	☐	☐	☐
c. Apply fluoride varnish	☐	☐	☐	☐	☐	☐
d. Place preventive fissure sealants	☐	☐	☐	☐	☐	☐
e. Demonstrate oral health maintenance (e.g. tooth brushing)	☐	☐	☐	☐	☐	☐
f. Take bitewing radiographs	☐	☐	☐	☐	☐	☐

Q2 As part of managing a child with the following presentation, I would:

	An occlusal, cavitated lesion with pulpal involvement in a deciduous tooth			An enamel-only proximal lesion in a first permanent molar		
	Never	Case dependent	Always	Never	Case dependent	Always
a. Apply fluoride varnish	☐	☐	☐	☐	☐	☐
b. Place preventive fissure sealants	☐	☐	☐	☐	☐	☐
c. Demonstrate oral health maintenance (e.g. tooth brushing)	☐	☐	☐	☐	☐	☐
d. Take bitewing radiographs	☐	☐	☐	☐	☐	☐
e. Carry out partial caries removal	☐	☐	☐	☐	☐	☐
f. Carry out complete caries removal	☐	☐	☐	☐	☐	☐
g. Place a stainless steel crown	☐	☐	☐	☐	☐	☐
h. Place a glass ionomer restoration	☐	☐	☐	☐	☐	☐
i. Carry out pulp therapy	☐	☐	☐	☐	☐	☐
j. Perform an extraction	☐	☐	☐	☐	☐	☐
k. Watch and wait	☐	☐	☐	☐	☐	☐

Q3 Please provide any explanation or comments for Questions 1 & 2 below:

Q4 The following members of my dental team are likely to:

Please tick all that apply

	Dental nurse	Extended duty dental nurse	Hygienist	Therapist	Dentist
a. Record caries risk	☐	☐	☐	☐	☐
b. Choose a recall interval based on caries risk	☐	☐	☐	☐	☐
c. Demonstrate oral health maintenance (e.g. tooth brushing, flossing)	☐	☐	☐	☐	☐
d. Apply fluoride varnish	☐	☐	☐	☐	☐
e. Place preventive fissure sealants	☐	☐	☐	☐	☐
f. Take bitewing radiographs	☐	☐	☐	☐	☐
g. Carry out partial caries removal	☐	☐	☐	☐	☐
h. Carry out complete caries removal	☐	☐	☐	☐	☐
i. Place a stainless steel crown	☐	☐	☐	☐	☐
j. Place a glass ionomer restoration	☐	☐	☐	☐	☐
k. Carry out pulp therapy	☐	☐	☐	☐	☐
l. Perform an extraction	☐	☐	☐	☐	☐

Q5 Please provide any explanation or comments below:

Section 2: Your views – VERSION 1: Record risk, apply fluoride varnish

Q1 In general, when I think about caries risk assessment:

	Strongly disagree						Strongly agree
a. I know how to classify each patient's caries risk	1	2	3	4	5	6	7
b. I know how to record each patient's caries risk	1	2	3	4	5	6	7
c. I know recording risk is advocated within current clinical guidance	1	2	3	4	5	6	7
d. I think recording caries risk assessment is strongly supported by evidence	1	2	3	4	5	6	7
e. Recording caries risk is not a priority for me	1	2	3	4	5	6	7
f. It is difficult for me to assess an individual patient's caries risk	1	2	3	4	5	6	7
g. I am confident I can correctly classify an individual patient's caries risk	1	2	3	4	5	6	7
h. Recording caries risk improves the quality of care I provide	1	2	3	4	5	6	7
i. I believe recording caries risk is important	1	2	3	4	5	6	7
j. I can easily remember to record caries risk	1	2	3	4	5	6	7
k. I think recording caries risk requires more time than I have during a routine consultation	1	2	3	4	5	6	7
l. I think recording caries risk is an efficient use of my time	1	2	3	4	5	6	7
m. My patient records have designated space to record caries risk	1	2	3	4	5	6	7
n. Recording caries risk is difficult for this practice to include in record management	1	2	3	4	5	6	7
o. I usually record caries risk	1	2	3	4	5	6	7
p. I believe recording caries risk is an important part of my professional role	1	2	3	4	5	6	7
q. I believe recording caries risk is an important part of the role of other dental team members	1	2	3	4	5	6	7
r. Caries risk recording is supported by my dentist colleagues at this/other practices	1	2	3	4	5	6	7

Additional file 2
Diagnostic questionnaire

Q2	In general, I think applying fluoride varnish twice a year for my child patients:	Strongly disagree						Strongly agree
	a. Is advocated within current clinical guidance	1	2	3	4	5	6	7
	b. Is strongly supported by evidence	1	2	3	4	5	6	7
	c. May have negative consequences for my patients	1	2	3	4	5	6	7
	d. May have negative consequences for me	1	2	3	4	5	6	7
	e. Is not a priority for me	1	2	3	4	5	6	7
	f. Will generally improve the oral health of a patient	1	2	3	4	5	6	7
	g. Has benefits which outweigh the costs	1	2	3	4	5	6	7
	h. Is important	1	2	3	4	5	6	7
	i. Prevents caries	1	2	3	4	5	6	7
	j. Is something for which I receive appropriate financial compensation	1	2	3	4	5	6	7
	k. Would increase in this practice if it was more financially rewarding	1	2	3	4	5	6	7
	l. Requires more time than I have during a routine consultation	1	2	3	4	5	6	7
	m. Requires more resources (e.g. staff, space, equipment) than I have available	1	2	3	4	5	6	7
	n. Is difficult for this practice to include in the record management system	1	2	3	4	5	6	7
	o. Is an important part of my professional role	1	2	3	4	5	6	7
	p. Is an important part of the role of other dental team members	1	2	3	4	5	6	7
	q. Is my responsibility	1	2	3	4	5	6	7
	r. Is supported by my dentist colleagues in this/other practices	1	2	3	4	5	6	7
	s. Is something children want	1	2	3	4	5	6	7
	t. Is something parents want for their children	1	2	3	4	5	6	7

Additional file 2
Diagnostic questionnaire

Section 2: Your views – VERSION 2: Use risk-based recall intervals, place fissure sealants

Q1 In general, I think selecting a follow up interval based on individual caries risk:

	Strongly disagree						Strongly agree
a. Is advocated within current clinical guidance	1	2	3	4	5	6	7
b. Is strongly supported by evidence	1	2	3	4	5	6	7
c. Will improve the quality of care I provide	1	2	3	4	5	6	7
d. Will generally improve the oral health of my patients	1	2	3	4	5	6	7
e. Is not a priority for me	1	2	3	4	5	6	7
f. Is something I can easily remember to do	1	2	3	4	5	6	7
g. Requires more time than I have during a routine consultation	1	2	3	4	5	6	7
h. Is an efficient use of my time	1	2	3	4	5	6	7
i. Is important	1	2	3	4	5	6	7
j. Is difficult to incorporate into the scheduling system at this practice	1	2	3	4	5	6	7
k. Is something I usually do	1	2	3	4	5	6	7
l. Is an important part of my professional role	1	2	3	4	5	6	7
m. Is reliant on other dental team members' contributions to caries risk assessment	1	2	3	4	5	6	7
n. Is reliant on my ability to correctly classify caries risk	1	2	3	4	5	6	7
o. Is something I am confident doing	1	2	3	4	5	6	7
p. Is supported by my dentist colleagues in this/other practices	1	2	3	4	5	6	7
q. Is something I know how to do	1	2	3	4	5	6	7
r. Is something I can easily explain to my patients	1	2	3	4	5	6	7
s. Is something about which I am comfortable answering patient questions	1	2	3	4	5	6	7

Additional file 2
Diagnostic questionnaire

**Q2** In general, I think placing preventive fissure sealants for my patients:	**Strongly disagree**						**Strongly agree**
a. Is advocated within current clinical guidance	1	2	3	4	5	6	7
b. Is strongly supported by evidence	1	2	3	4	5	6	7
c. Is not a priority for me	1	2	3	4	5	6	7
d. Will generally improve the oral health of patients	1	2	3	4	5	6	7
e. May have negative consequences for my patients	1	2	3	4	5	6	7
f. Has benefits which outweigh the costs	1	2	3	4	5	6	7
g. Prevents the development of caries	1	2	3	4	5	6	7
h. Is important	1	2	3	4	5	6	7
i. Is something for which I receive appropriate financial compensation	1	2	3	4	5	6	7
j. Would increase in this practice if it was more financially rewarding	1	2	3	4	5	6	7
k. Requires more time than I have during a routine consultation	1	2	3	4	5	6	7
l. Requires more resources (e.g. staff, space, equipment) than I have available	1	2	3	4	5	6	7
m. Is an important part of my professional role	1	2	3	4	5	6	7
n. Is an important part of the role of other dental team members	1	2	3	4	5	6	7
o. Is supported by my dentist colleagues in this/other practices	1	2	3	4	5	6	7
p. Is something children want	1	2	3	4	5	6	7
q. Is something parents want for their children	1	2	3	4	5	6	7
r. Is something adults want	1	2	3	4	5	6	7
s. Is my responsibility	1	2	3	4	5	6	7
t. Is something I find difficult to know when is indicated	1	2	3	4	5	6	7
u. Is something I find difficult to do	1	2	3	4	5	6	7

Section 2: Your views – VERSION 3: Demonstrate OH maintenance, take bitewing radiographs

Q1 In general, I think demonstrating oral health maintenance (e.g. tooth brushing, flossing):

	Strongly disagree						Strongly agree
a. Is advocated within current clinical guidance	1	2	3	4	5	6	7
b. Is strongly supported by evidence	1	2	3	4	5	6	7
c. Is an effective way for me to encourage good oral health maintenance for my patients	1	2	3	4	5	6	7
d. Is something I find difficult to do	1	2	3	4	5	6	7
e. Is not a priority for me	1	2	3	4	5	6	7
f. Will generally improve the oral health of a patient	1	2	3	4	5	6	7
g. Prevents caries	1	2	3	4	5	6	7
h. Is something I am able to demonstrate effectively	1	2	3	4	5	6	7
i. Has benefits which outweigh the costs	1	2	3	4	5	6	7
j. Is important	1	2	3	4	5	6	7
k. Is something for which I receive adequate financial compensation	1	2	3	4	5	6	7
l. Would increase in this practice if it was more financially rewarding	1	2	3	4	5	6	7
m. Requires more time than I have during a routine consultation	1	2	3	4	5	6	7
n. Requires more practice resources (e.g. staff, space, equipment) than I have available	1	2	3	4	5	6	7
o. Makes me feel uncomfortable	1	2	3	4	5	6	7
p. Is an important part of my professional role	1	2	3	4	5	6	7
q. Is an important part of the role of other dental team members	1	2	3	4	5	6	7
r. Is supported by my dentist colleagues at this/other practices	1	2	3	4	5	6	7
s. Is something children and parents I see want	1	2	3	4	5	6	7
t. Is something adults I see want	1	2	3	4	5	6	7
u. Is something I am responsible for ensuring is provided to patients	1	2	3	4	5	6	7

Additional file 2
Diagnostic questionnaire

Q2 In general, for the following patients, I think taking bitewing radiographs:

	Child with deciduous teeth							Child with permanent and deciduous teeth							An adult						
	**Strongly disagree**						**Strongly agree**	**Strongly disagree**						**Strongly agree**	**Strongly disagree**						**Strongly agree**
a. Is advocated within current clinical guidance	1	2	3	4	5	6	7	1	2	3	4	5	6	7	1	2	3	4	5	6	7
b. Is strongly supported by evidence	1	2	3	4	5	6	7	1	2	3	4	5	6	7	1	2	3	4	5	6	7
c. Is something I find difficult to do	1	2	3	4	5	6	7	1	2	3	4	5	6	7	1	2	3	4	5	6	7
d. Improves my ability to manage caries	1	2	3	4	5	6	7	1	2	3	4	5	6	7	1	2	3	4	5	6	7
e. Does not produce images of consistently adequate quality	1	2	3	4	5	6	7	1	2	3	4	5	6	7	1	2	3	4	5	6	7
f. Is not a priority for me	1	2	3	4	5	6	7	1	2	3	4	5	6	7	1	2	3	4	5	6	7
g. May have negative consequences for my patients	1	2	3	4	5	6	7	1	2	3	4	5	6	7	1	2	3	4	5	6	7
h. May have negative consequences for me	1	2	3	4	5	6	7	1	2	3	4	5	6	7	1	2	3	4	5	6	7
i. Does not influence the way I manage caries	1	2	3	4	5	6	7	1	2	3	4	5	6	7	1	2	3	4	5	6	7
j. Is important	1	2	3	4	5	6	7	1	2	3	4	5	6	7	1	2	3	4	5	6	7
k. Is something for which I receive appropriate financial compensation	1	2	3	4	5	6	7	1	2	3	4	5	6	7	1	2	3	4	5	6	7
l. Would increase in this practice if it was more financially rewarding	1	2	3	4	5	6	7	1	2	3	4	5	6	7	1	2	3	4	5	6	7
m. Requires more time than I have during a routine consultation	1	2	3	4	5	6	7	1	2	3	4	5	6	7	1	2	3	4	5	6	7
n. Is supported by my dentist colleagues in this/other practices	1	2	3	4	5	6	7	1	2	3	4	5	6	7	1	2	3	4	5	6	7
o. Is something children want	1	2	3	4	5	6	7	1	2	3	4	5	6	7	1	2	3	4	5	6	7
p. Is something parents/adults want	1	2	3	4	5	6	7	1	2	3	4	5	6	7	1	2	3	4	5	6	7
q. Makes me feel anxious	1	2	3	4	5	6	7	1	2	3	4	5	6	7	1	2	3	4	5	6	7
r. Makes my patients feel anxious	1	2	3	4	5	6	7	1	2	3	4	5	6	7	1	2	3	4	5	6	7
s. Would have helped me identify past caries at an earlier stage	1	2	3	4	5	6	7	1	2	3	4	5	6	7	1	2	3	4	5	6	7
t. Is difficult to include in the record management system	1	2	3	4	5	6	7	1	2	3	4	5	6	7	1	2	3	4	5	6	7
u. Is difficult to know when to do	1	2	3	4	5	6	7	1	2	3	4	5	6	7	1	2	3	4	5	6	7

Section 3: About this practice

Please answer the following questions in relation to the practice/clinic where you received this questionnaire.

**Q1**	Is this the only practice where you work?	Yes ☐ No ☐
**Q2**	Is this a vocational training practice?	Yes ☐ No ☐
**Q3**	How many sessions (0.5 days) per week do you work at this practice?	☐ Sessions
**Q4**	What is your approximate list size at this practice?	☐ Patients
**Q5**	What approximate percent of your patients at this practice are under age 16?	☐ Percent
**Q6**	What is the approximate ratio of NHS to private pay patients at this practice?	: NHS:Private
**Q7**	How many other dentists are at this practice?	☐ Dentists
**Q8**	Does this practice employ a dental hygienist?	Yes ☐ No ☐
**Q9**	Does this practice employ a dental therapist?	Yes ☐ No ☐
**Q10**	Does this practice employ an extended duty dental nurse?	Yes ☐ No ☐
**Q11**	Does this practice employ a practice manager?	Yes ☐ No ☐
**Q12**	If there is no practice manager, does someone else fill the same role?	Yes ☐ No ☐ N/A ☐
	Who (*e.g. dental nurse, GDP*)?	
**Q13**	Is this practice, or has it ever been, a Childsmile practice?	Yes ☐ No ☐
**Q14**	When did this practice become a Childsmile practice?	☐ Month/Year
**Q15**	Does this practice have computerized records?	Yes ☐ No ☐ Mix ☐
	What software system?	
**Q16**	In this practice, is there an established system other than a complaints log to gather patient feedback? *(e.g. satisfaction surveys, feedback forms, suggestion box, etc)*	Yes ☐ No ☐
**Q17**	In this practice, is there routine (at least every 2 years) evaluation or quality assurance of caries related prevention and/or management? *(e.g. internal data review, radiograph audit, guidance review, etc)*	Yes ☐ No ☐

Q18 In general, is available guidance for the prevention and management of caries applicable in this practice:

	**Strongly disagree**						**Strongly agree**
a. For care of children?	1	2	3	4	5	6	7
b. For care of adults?	1	2	3	4	5	6	7

Section 4: About you

Please answer the following questions in relation to all the practices/clinics where you work.

Q1 How would you describe yourself?

☐ Principal ☐ Associate ☐ Salaried ☐ Vocational trainee ☐ Other: ____________________

Q2 Are you currently a vocational trainer? Yes ☐ No ☐

Q3 How many sessions (0.5 days) per week do you work in total? ☐ Sessions

Q4 What is your approximate list size for all the practices where you work? ☐ Patients

Q5 In which setting do you work the most?

☐ General Dental ☐ Community Dental ☐ Salaried GDS ☐ Private ☐ Other: ____________________

Q6 Do any of the other practices where you work claim for preventive dental services delivered to children 2-5 years old? Yes ☐ No ☐

Q7 Were any of the other practices where you work Childsmile demonstration practices (before October 2011)? Yes ☐ No ☐

Q8 Please tell us your age: ☐ Years

Q9 Please tell us your gender: Male ☐ Female ☐

Q10 Please tell us when you qualified: ☐ Month/Year

Q12 Please provide any additional comments in relation to any part of this questionnaire:

☐

Thank you very much for your time completing this questionnaire.
Please return by 19 September 2012 in the Freepost envelope marked SURVEY.
