# Supplementary material for: Patient-, organization-, and system-level barriers and facilitators to preventive oral health care: a convergent mixed-methods study in primary dental care
Source: Implement Sci. 2016 Jan 12;11:5. doi: 10.1186/s13012-015-0366-2 (PMC4710040; doi:10.1186/s13012-015-0366-2)
Supplement: Supplementary file 3 — Case study and patient feedback data collection tools. Qualitative data collection tools. [file 13012_2015_366_MOESM3_ESM.pdf]

Additional file 3
Case study and patient feedback data collection tools

Case study observation guide

1. Observe and describe the content and delivery of care within case study practices in relation to:
    a. General care (including history taking, updating records, advice or treatment, etc)
    b. Care in relation to the six study behaviours (add list here or upfront as a reference for observer).
        i. Assess caries risk for children/adults
        ii. Document caries risk for children/adults
        iii. Apply fluoride varnish for children/adults
        iv. Place fissure sealants for children/adults
        v. Demonstrate activities like flossing or tooth brushing for children/adults
        vi. Take bitewing x-rays for children/adults
2. Observe and describe the factors or events which:
    a. Appear to influence whether (and how) preventative care in relation to the six study behaviours is delivered during the consultation,
    b. Appear to make delivery of those preventative behaviours easier or more difficult.
3. Describe the practice context of care delivery (*working list to guide field notes*)
    a. Space
        i. Neighbourhood
        ii. Exterior
        iii. Waiting room
        iv. Reception
        v. Staff room
    b. Time
        i. Open times
        ii. Day of week
        iii. Morning/afternoon session
    c. Movement
        i. Reception
        ii. Paperwork
        iii. Waiting room
        iv. Into surgery
    d. Information
        i. Paperwork
        ii. Posters
        iii. Fliers
        iv. Other printed material
    e. Communication
        i. Within groups (e.g. reception, dental nurses, dentists)
        ii. Across groups
        iii. Outside surgery
        iv. Inside surgery

Additional file 3
Case study and patient feedback data collection tools

Dental team member interview topic guide

INTERVIEWEE ROLE: ____

INTERVIEW METHOD: ____

1. Please describe the routine care you'd provide to prevent caries for:
    a. A child
    b. An adult
2. Please describe the routine care you'd provide to manage caries for:
    a. A child
    b. An adult
3. When you think about guidance such as '*Oral Health Assessment and Review*' or *'Prevention and Management of Child Caries '*:
    a. How easy is it to understand the guidance? Is there anything that could be done to make the guidance easier to understand?
    b. What do you understand as being the key recommendations?
        i. For children
        ii. For adults
    c. How strong do you think the evidence is for the guidance/recommendations? Is there anything that would give you more confidence in the guidance?
4. When you think of guidance like '*Prevention and Management of Child Caries*' or '*Oral Health Assessment*':
    a. What do you think the purpose is? Is that what the purpose should be, in your opinion?
    b. How relevant do you think the guidance is to your role as _______________?
    c. Does the guidance help you give preventive care or advice to patients? Why or why not?
    d. How important do you think it is to address prevention in guidance? Are recommendations for prevention as important as recommendations for treatment?
5. When new or updated guidance is published by an organization like SDCEP or NICE:
    a. How and when do you find out about it? Yourself?
        i. Prompt: Do you look for it yourself or does it come to you? From within the practice or another outside source such as GDC or SDCEP?
    b. How do others in your practice (your practice in general) find out about it?
    Prompt: How is it delivered? Format? Who gets it? How is it passed along within the practice (meetings, designated person, etc)?
    c. How are the recommendations put into practice? E.g. twice yearly fluoride varnish, risk based recall, etc
    d. Do you have a role in this process? What is it?
    e. How do you/others in your practice know when the guidance recommendations are being followed? Is this monitored in any way?

6. This study is generally interested in the prevention and management of caries. We're also interested in specific recommendations within caries guidance – these are recording caries risk in patient records, basing recall intervals on caries risk, applying fluoride varnish, placing fissure sealant, demonstrating oral health maintenance activities such as tooth brushing and flossing, and using bitewing radiographs. Thinking generally or in relation to these recommendations:
    a. How do you discuss/deliver guidance recommendations with:
        i. Children/parents?
        ii. Adults?
    b. To what extent do you think you and your practice are able to implement general recommendations in current guidance (e.g. Child Caries, Oral Health Assessment)?
    c. To what extent do you think you and your practice are able to implement specific recommendations such as the ones I mentioned before (e.g. recording risk, risk based recall, fluoride varnish, fissure sealant, oral health demonstration, bitewing x-rays)?
7. Thinking about the day to day care you provide for the prevention and management of caries:
    a. What factors influence the extent to which you implement current guidelines? (Probe: barriers and facilitators)
        i. For children?
        ii. For adults?
    b. Which of the factors you've mentioned *most* influence your ability to implement current guidelines?
    c. *Patients*
        i. How much do your individual patients influence the extent to which you can deliver caries guidance/discuss recommendations with them? Probe: behaviour, time, education, lifestyle, expectations or attitudes
        ii. How important do you think prevention and management caries is to your patients? Are there other issues of greater priority to patients?
        iii. What do you think your patients expect from you in relation to prevention or management of caries? How do you think you can meet your patients' expectations? (Prompt: barriers, facilitators)
    d. *Practice*
        i. In terms of your role as ________, what is your involvement in caries prevention and management? Do you think this is what it should be?
        ii. What influences your ability to prevent or manage caries for children and adults? E.g. individual level factors (skills, knowledge, confidence), practice level factors (leadership, delegation, communication, time, space, personnel, equipment, etc) (Prompt: Which factors are barriers and which are facilitators?)
    e. *Health care system*
        i. What aspects of the health care system, from the board to national level, influence your ability to prevent or manage caries for children and adults? E.g. remuneration, education and training, public health initiatives like Childsmile, performance targets at a board or national level (e.g. Heat Targets) etc.
        Probe: barriers and facilitators
        ii. From the point you decided to become a _______ until now, how has your education and training prepared you to:
            1. Provide preventive care for children and adults?
            2. Integrate guidance recommendations into your routine practice?

8. What could be done (or what change would be needed /what would have to happen) to increase the extent to which you/ your practice is able to deliver preventative care in line with current guidelines:
    a. Patient level?
    b. Practice level? (Prompt: leadership, delegation, communication, time, space, personnel, equipment, etc.)
    c. Systems level? (Prompt: remuneration, education and training, public health initiatives like Childsmile, performance targets at a board or national level)
    d. What do you think would make the biggest difference in helping you provide preventive care?
9. Do you have any additional comments or questions for us?

Additional file 3
Case study and patient feedback data collection tools

Patient focus group guide

1. How important is the health of your mouth and teeth in relation to your health overall?
    - Why oral health is more/less/the same importance as overall health
    - Oral health as a priority
2. What do you do to keep your/your children's teeth and mouth healthy?
    - To avoid decay from starting
    - To stop decay from getting worse
        - Prompt – Do you know what options there are to take care of any decay you/your children might have? (e.g. FS, stainless steel crowns, restoration v. extraction)
    - Are you willing to do more to avoid or treat decay in some parts of your/child's mouth than others?
        - Prompt – Do front teeth matter more than back teeth? Does pain matter more than no pain? Do baby teeth matter?
    - Can decay be avoided or does it happen no matter what you do?
3. When you think about the things you could do to look after the health of your teeth and mouth (or if you are responsible for children, their teeth and mouth) are there any that you find especially difficult to do or think you could do better? What makes them difficult?
        - Prompts for all:
            - Time, routine
            - Care responsibilities
            - Supplies, equipment
            - Knowledge
            - Skills/capabilities
            - Feedback/reinforcement/outcomes
            - Costs/benefits
    - Problems or difficulties associated with caring for your teeth at home
    - Problems or difficulties with getting treatment or advice
    - Ways to make it easier to care for teeth
4. Thinking about the information and advice you've received about caring for your/your children's teeth what makes it easier or harder to follow that advice?
        - Prompt – When and what was the most recent information you got? Who gave it to you or where did it come from?
    - Frequency of information
    - Usual source of advice – dentist, dental practice, internet, family/friends, etc
    - Usual content – skills/technique, equipment/supplies, diet/drinks/nutrition/sugar, etc
    - Type/format of information – advice, leaflets, sugar diaries, etc
    - Who gives you the information, how it is delivered – hand out, discussion, demonstration, etc
    - What type of information is the most helpful?
        - Prompt – Frequency, format, who and how delivered
        - Prompt – Information you ask for versus information just given to you
    - How do you use the information you get? How do you decide what advice to take?

5. What do you think about advice for avoiding or reducing tooth decay you get from your dentist, hygienist, dental nurse, or other sources?

    - Information/advice makes sense, meets individual needs/conditions
    - Dentist/people in the practice help think of ways to make it easier to follow advice
        - Prompt – Do you think your dentist or other people in the practice know what the best things for you to do are?
        - Probe – What has led to this opinion?
    - Dentist and other people at dental practice seem to have time to give advice you want/need
    - Importance of advice about avoiding decay
        - At a routine check up
        - At a visit for treatment
            - Prompt – Does it matter who gives you the advice?
            - Prompt – Do you just get advice or do you have to ask for it? Should you have to ask or should it just be given to you? Do you have a preference?
    - Advice received met expectations and was helpful
        - Prompt – If yes, how? If no, why?
    - Additional advice or information which would help in taking care of mouth and teeth

6. If you're a parent, or if you have family and close friends with children, and you think about the things we've just discussed, what do you think makes it easier or harder to keep children's teeth healthy and avoid decay?

    - Difficulties/barriers
        - Prompt – At home?
        - Prompt – At the dentist's?
    - Things that make it easier
        - Prompt – At home?
        - Prompt – At the dentist's?

7. Is there anything else you can think of that would help you or your children to take care of your teeth, avoid decay, or take care of any decay?

    - At home
    - At dentist practice
    - In community – schools, health centres, home visitors, etc
    - In products and advertising – oral health care products, food/drinks

8. Is there anything else you'd like to add?

Additional file 3
Case study and patient feedback data collection tools

Patient telephone interview guide

About you:

1. What do you currently do to keep your mouth and teeth healthy?
    a. Do you think you should be doing anything differently? If so, what? Why don't you do it? What makes it difficult to do? What could make it easier?
    b. Who do you think has the most important role in keeping your mouth and teeth healthy, your dentist or you?
2. What do you expect your dentist to tell you about keeping your mouth and teeth healthy?
    a. What have you really liked or found useful in the past?
    b. What have you disliked or found unhelpful?
3. How do you get information and advice about keeping your teeth healthy?
    a. Do you need to ask for information or advice or do you just get it?
    b. Do you ask your dentist questions? What kind of questions do you ask?
    c. When was the most recent time you got advice?
    d. What topics did it cover? Was it just about tooth brushing or did it include other things like flossing and diet?
    e. What was it like? Helpful, boring, confusing, annoying, repetitive, irritating, etc? Why?
4. There are many people who might also give you advice about your oral health – hygienists, dental nurses, community nurses, your GP – do you think:
    a. It makes a difference who advises you about your oral health in terms of how likely you are to follow that advice?
    b. It helps to get advice from multiple sources? Or does it make it more confusing as to what you are supposed to do to keep your mouth and teeth healthy?
5. There are several things dentists should do to help prevent tooth decay, has your dentist ever done these things for you?
    a. Fluoride varnish
    b. Fissure sealants
    c. Shown you how to clean your teeth or your child's teeth (tooth brush, floss)
    d. Taken dental x-rays
6. Have you ever commented or given any kind of feedback to your dentist about the kind of care they give you? Either good or bad?
7. What do you think is the most important thing for keeping your mouth and teeth healthy?

About your children:

1. Do you have children? How old are they? Have they ever had any decay in their teeth?
2. What do you generally do to keep their mouths and teeth healthy?
    a. How involved are you in taking care of their teeth? Brush their teeth for them, supervise, leave it to them?
    b. Do you think you should be doing anything differently? If so, what? Why don't you do it? What makes it difficult to do? What could make it easier?

   c. Does your dentist think you should be doing anything differently? If so, what? Why don't you do it? What makes it more difficult to do? What could make it easier?
   d. Who do you think has the most important role in keeping your child's mouth and teeth healthy? Your dentist, your child, or you?

3. What do you expect your dentist to tell you about keeping your child's mouth and teeth health?
   a. What have you really liked or found useful in the past?
   b. What have you disliked or found helpful?
4. How do you get information and advice about keeping your child's teeth healthy?
   a. Do you need to ask for information or advice or do you just get it?
   b. Do you ask your dentist questions? What kind of questions do you ask?
   c. When was the most recent time you got advice?
   d. What topics did it cover? Was it just about tooth brushing or did it include other things like flossing and diet?
   e. What was it like? Helpful, boring, confusing, annoying, repetitive, irritating, etc? Why?
5. When your children go to the dentist:
   a. Are you comfortable discussing your child's care with their dentist? Why or why not?
   b. Are you comfortable discussing the health of your child's mouth and teeth with your dentist? Why or why not?
   c. How likely are you to refuse something your dentist recommends such as fluoride varnish if:
      i. Your child really doesn't want to have it done?
      ii. You don't like the sound of it or don't feel you have enough information to make a decision?
6. There are many people who might also give you advice about your children's oral health – hygienists, school nurses, community nurses, their GP – do you think:
   a. It makes a difference who advises you about your child's oral health in terms of how likely your children or you are to follow that advice?
   b. It helps to get advice from multiple sources? Or does it make it more confusing as to what your children and you are supposed to do to keep their mouth and teeth healthy?
7. There are several things dentists should do to help prevent tooth decay, has your dentist ever done these things for your child/children?
   a. Fluoride varnish
   b. Fissure sealants
   c. Shown you how to clean your teeth or your child's teeth (tooth brush, floss)
   d. Taken dental x-rays
8. What do you think is the most important thing for keeping your child's mouth and teeth healthy?
9. Is there anything else you'd like to add? Do you have any questions?
