# Supplementary material for: Patient-, organization-, and system-level barriers and facilitators to preventive oral health care: a convergent mixed-methods study in primary dental care
Source: Implement Sci. 2016 Jan 12;11:5. doi: 10.1186/s13012-015-0366-2 (PMC4710040; doi:10.1186/s13012-015-0366-2)
Supplement: Supplementary file 4 — Dental team member interview and patient feedback coding guides. Qualitative TDF coding guides. [file 13012_2015_366_MOESM4_ESM.pdf]

Additional file 4
Dental team member interview and patient feedback coding guides

Additional file 4.1 – Dental team member interview coding guide

Theoretical Domain	Related constructs	Guiding Notes	Sample Quotes
Knowledge (K)	Knowledge<br><br>Knowledge about the outcome/ scientific rationale<br><br>Schemas and mindsets and illness representations<br><br>Procedural knowledge	Explicitly stating they have knowledge, awareness or familiarity (or none) about guidance document, evidence or target behaviour.<br><br>Expression of confidence or trust in the guidance document, evidence or target behaviour	➢ we understand the information and we can like, we take it and I may know everything [Dental Nurse]<br>➢ I don’t know what the research is for fluoride varnish. When I was a student not all toothpastes were fluoridated so we were taught fluoride varnish techniques etc. but now virtually all toothpastes are fluoridated how many additional fluoride applications per year is the correct amount, I don’t know [Dentist]<br>➢ there’s loads of different tools that you get out there that you can use to clean in between your teeth [Dental Nurse]<br>➢ I would say I would probably have to look at the evidence a little bit more myself [Dentist]<br>➢ It’s just mostly about updating our information, that’s really what it’s for, just keeping you up to date with everything because things change so regular, it’s just basically about keeping you updated with everything so it is. [Dental Nurse]

Additional file 4
Dental team member interview and patient feedback coding guides

Skills (S)	Skills<br><br>Competence/ability<br><br>Skill development/practice<br><br>Interpersonal skills<br><br>Coping strategies<br><br>Skills assessment	Explicitly stating the ability to do something/an observable skill.<br><br>Only code here if it is actually observable and not a perception of a skill (which makes it a capability)	Nothing coded to this domain
Beliefs about capabilities (CA)	Self efficacy<br><br>Perceived competence<br><br>Control of behaviour<br><br>Control of environment (material & social)<br>Perceived behavioural control<br><br>Self esteem<br><br>Self confidence/ professional confidence<br><br>Optimism/pessimism<br><br>Empowerment	Perceived ease/difficultly in carrying out the behaviour.<br><br>Perceptions about their own or others competence and confidence to do a behaviour	➢ 'I see my education as an undergraduate did help me quite well to treat caries and also prevent caries. [Dentist]<br>➢ 'Yes, because I think we can, we can like pass it on really good because… I think we can relay it on to them [Dental Nurse]<br>➢ I like to think I'm quite thorough, I don't miss any cavities or anything [dentist]<br>➢ I feel well equipped to deal with caries and do early intervention [Dentist]

Beliefs about consequences (CO)	Outcome expectancies<br><br>Anticipated regret<br><br>Unrealistic optimism<br><br>Appraisal/evaluation/review<br>Attitudes<br><br>Beliefs<br><br>Contingencies<br><br>Reinforcement/punishment/consequences<br><br>Incentives/rewards<br><br>Salient events, critical incidents, sensitisation<br><br>Characteristics of outcome expectancies include – physical, social, emotional; proximal/distal, valued/not valued, likely/unlikely, risk/reward; salient/not salient	Beliefs, attitudes, and expectations regarding the outcomes and consequences of the behaviour (either positive or negative) to both self and others.<br><br>This can sometimes be confused with SI, need to be clear if it is the consequences of the behaviour on the patient/dentist (i.e. Consequences) or the influence of others' behaviour/attitudes on the professional (i.e. Social influence)<br><br>Things like a rapport with the patient comes under SI as it makes it easier to do the behaviour (it is not a consequence of the behaviour itself, and therefore does not come under CO.)	➢ 'I don't think they, well not all patients expect it from you, but I think they appreciate it when they're given it' [Dental Nurse]<br>➢ they're in there to get their teeth fixed, get their mouth better obviously, and you're giving them the advice to do this [Dentist]<br>➢ because if you prevent it then you don't even need to like deal with the whole other situation. So if you can stop it before it even happens then using, like basically that helps them in other areas of life as well, because obviously it's Ok to come in and get a filling, but if you can tell them how to stop getting them [Dental Nurse]<br>➢ 'I will do it in cases where I think it might be beneficial' [Dentist]<br>➢ I think the amount of cases of nursing multiple caries I have seen it's definitely, definitely decreasing because mothers are definitely getting the information they need now rather than leaving it too late until the child is two or three [Dentist]<br>➢ The cost/benefit isn't clear to me at all. And the evidence, again, I don't know. I'd understand the logic of fissure sealant but is the benefit from fissure sealant so great as to allow the use of that much time? [Dentist]

Motivation, goals, intent (MI)	Intention: stability/certainty<br><br>Goals (autonomous/controlled)<br><br>Goal target setting<br><br>Proximal and distal goals<br><br>Intrinsic motivation<br><br>Commitment<br><br>Stages of change<br><br>Goal priority	Want or desire to carry out the behaviour.<br><br>Prioritising it above other behaviours.<br><br>Intention to perform the behaviour	➢ 'Obviously we're wanting to give them any advice or anything possible to obviously try and prevent this. So anything like really is really important to say to them and give them advice on' [Dental Nurse]<br>➢ I'm not going to interfere with that [Dentist]
Memory, attention, decision making (MAD)	Memory<br><br>Attention<br><br>Attention control<br><br>Decision making	Attention/remembering to perform a behaviour	➢ No, sometimes, sometimes you remember, but obviously you're not going to remember with everybody' [Dental Nurse]

Environmental context, resources, constraints (E)	Resources (material, temporal, spatial, personnel): availability<br><br>Resource management<br><br>Environmental stressors<br><br>Person x environment interaction<br><br>Knowledge of task environment	Factors relating to the surgery which influence whether or not a behaviour is being carried out (e.g. availability of time, space, staff, equipment etc.)<br><br>If there is a high burden of caries this is coded here as this has an impact on time available to do prevention	➢ we've ordered in leaflets for the waiting room just for patients, and they do like go down, you do see people taking them, so I think it does help [Dental Nurse]<br>➢ If the surgery was a wee bit bigger you could probably get round to the patient and speak it's really hard to do, it is really quite wee, so you're actually squeezing passed the dentist to do stuff [Dental Nurse]<br>➢ I'd say a wee bit more time. Like if you had longer for each patients for appointments I would say it'd be a lot easier, because sometimes if they're in for their fillings and things like that then you've got half an hour, you've done your fillings and you kind of, "I've done this, that, there" and then they go. So if you did have maybe a wee bit longer to spend time with patients you could go over all this a lot more I would say, yes definitely [Dental Nurse]<br>➢ 'obviously their appointments times sometimes are no long enough' [Dental Nurse]<br>➢ is like classed as a deprived area, so you are getting a lot of patients that are coming in from the kind of backgrounds that are, that's like really they're going to have caries really [Dental Nurse]

Social and Professional Role and Identity (SPI)	Identity<br><br>Professional identity/boundaries/role<br><br>Group/social identity<br><br>Social/group norms<br><br>Alienation<br><br>Organizational commitment	Professional identity (role, responsibilities boundaries, obligations)<br><br>Perception of own role within context of the practice/dental team (e.g. dental nurses feeling disempowered)<br><br>The point with this is that it is things intrinsic to the person, their perception of themselves as a particular professional role	➢ 'any questions asked, we're willing to answer them, so we don't mind people asking us questions really' [Dental Nurse]<br>➢ the dentist has probably already said to them, so we would be again just probably repeating it [Dental Nurse]<br>➢ the dentist would normally do obviously all the rest of the talking and the rest of the work [Dental Nurse]<br>➢ I see myself as primarily as a repairer of teeth. I feel prevention should be done in the communities [Dentist]<br>➢ I think sometimes it's more difficult than others obviously because you're not seeing inside their mouth, when they come for a check up, the dentist is doing the examination and you're charting, so it's not like I can turn round and say, "Well you're going to need two fissure sealants", obviously because I'm not at the clinical side doing it. So really I just feel that that's the dentist… [Dental Nurse]

Social Influences (SI)	Social support: inter-personal, professional, organizational, society/community<br><br>Social/group norms<br><br>Organizational climate, culture<br>Organizational development<br>Organizational commitment<br><br>Management, change management<br><br>Resource management<br><br>Leadership<br><br>Teamwork, conflict, competing demands/roles<br><br>Supervision, feedback<br><br>Learning and modelling<br><br>Champions, role models<br>Professional boundaries, roles<br><br>Inter-group conflict<br><br>Social comparisons<br><br>Negotiation<br><br>Group/social identity, conformity, alienation<br><br>Social pressure<br><br>Power, hierarchy	Views & behaviour from people from out with the practice (e.g. patients, Childsmile) which alters how a behaviour is done (or not done).<br><br>Other people expecting or not expecting the behaviour.<br><br>Other people influencing (facilitating or inhibiting) the behaviour<br><br>Observation of the behaviour in others influencing own behaviour<br><br>In contrast to SPI, this domain includes things external to the person, not their perceptions of their role but other people's attitudes and behaviour having an effect on the person's ability to carry out the behaviour.<br><br>So far the coding has identified social influence mainly in terms of the influence of the patients rather than management etc.	➢ 'I think, well normally they'd ask, if they've asked for it, then I think that's easier obviously' [Dental Nurse]<br>➢ 'they'll talk about it but quite often won't follow through. You can see that same child two weeks later somewhere else and they're swallowing Irn Bru' [Dentist]<br>➢ I'm generally led a little bit by gut and how I think the patient's responding – especially the kind of fluoride and advice kinda side of it, I'm a bit more patient-led and things [Dentist]<br>➢ But we've gotten varnishes recently because the Duraphat we were finding that children didn't like the taste so much and they were spitting it out so we got different flavours like caramel, melon, cherry, like all the nicer flavours so children tend to respond to them a little bit better [Dentist]<br>➢ and we do have some kids that you've given it to numerous times and they take none of it on board and they don't change anything (overspeaking) you can give them tooth brushes, toothpaste, everything away with them and they never seem to use it. It just depends whether they actually listen to what you say or whether they don't [Dental Nurse]

Emotion (Em)	Affect: positive, negative<br><br>Stress<br><br>Anticipated regret<br><br>Fear<br><br>Overload, fatigue, burn-out<br><br>Perceived threat<br><br>Anxiety, depression	Emotional benefit /risks in doing a behaviour, such things as indifference, there being no reward for doing behaviour	➢ 'you just feel it's a waste of time trying to help them and giving them advice. But if somebody is willing to listen, take the advice on board, then obviously you enjoy giving them advice and things like that' [Dental Nurse]<br>➢ That always makes you feel a little bit better and like you are getting through to them [Dentist]
Behavioural Regulation (BR)	Goal, target setting<br>Implementation intention<br><br>Moderators of intention-behaviour gap<br><br>Action planning<br><br>Generating alternatives<br><br>Self-monitoring<br><br>Prioritizing<br><br>Feedback<br><br>Project management<br><br>Barriers and facilitators	Ways of working that make it easier to carry out the intended behaviour at either the individual or organisational level.<br><br>Things people do to help themselves perform the behaviour/make the behaviour easier<br><br>Routines and systems (formal or informal) associated with the performance or modification of a behaviour<br><br>Strategies to facilitate and support behaviour, modify an existing behaviour, or introduce a new behaviour	➢ I would generally read it and because we don't have a designated Childsmile nurse at the moment I would probably do most of the stuff myself but there's another dentist here that we would chat about it and say, "What will we do?" [Dentist]<br>➢ obviously you do need to measure things and you do need to see are you doing the right thing and are there things we can improve [Dentist]<br>➢ And I've stopped doing fluoride drops for some years when the first evidence was coming out that topical application is much more effective [Dentist]

Nature of the Behaviour (NB)	Routine, automatic, habit<br><br>Breaking a habit<br><br>Direct experience, past behaviour<br><br>Stages of change	A description of what the person generally, currently tends to do;	➢ So adults, if a new patient was to come for a check-up we usually, again, categorise if they have low, medium or high caries risk' [Dentist]<br>➢ we'll just tell them like basically, break it down into simple like "The sugar's been eating away at your teeth and it's going to cause you pain if you don't get it done", and you just basically tell them, then we break it down further and say "You need to watch your teeth' [Dental Nurse]<br>➢ we do fissure seals as well actually, that's another thing for all kids between six and seven, we'll just assess them for fissure sealants. If they're part erupted I'll leave it for another six months but generally they'd all get fissure seals done which generally prevents decay [Dentist]<br>➢ I tend to not do as many fissure sealants as when I was first qualified [Dentist]<br>➢ It tends to be new patients. Or if they haven't been in for a little while. Or like with a previous patient I'll ask like, "Do you know how to floss? Have you been shown how to floss?" [Dentist]
**Coding notes:**			
1. Don't code if they are talking about decontamination guidance as that is not what we are interested in<br>2. Code for current practice not talking about the way things have been done in past or ideas for the future (these can go in ideas for improvement node)			

Additional file 4
Dental team member interview and patient feedback coding guides

Additional file 4.2 – Patient feedback coding guide

**Behaviours**	
BWR	Bitewing radiographs
FS	Fissure sealant
FV	Fluoride varnish
DOHM	Demonstration of oral health maintenance – demonstrated oral health maintenance by a health care professional, either on a model or on the patient
OHI	Oral health instruction – specific advice about oral health maintenance without demonstration
OHM	Oral health maintenance – patients' routines, behaviours for keeping teeth/mouth healthy
FB	Feedback – feedback patients have given to their dentist or other health care providers
Adv	Advice – general information and recommendations patients receive from the dentist or other health care providers
**Domains**	
K	Knowledge – patient knowledge
Co	Consequences – beliefs about benefits of care, barriers/facilitators to care
Em	Emotions – patient feelings
SI	Social influence – what patients expect in going to the dentist or other health care providers
SPI	Social professional role and identity – Patient perceived roles and responsibilities of health care professionals (both oral and general health)
NB	Nature of the behaviour – descriptions of oral health care and activities
**Additional**	
Imp	Improvement – patient suggestions for improvement
PR	Personal responsibility – patient perceptions of their role/responsibility for oral health
