# Supplementary material for: Patient-, organization-, and system-level barriers and facilitators to preventive oral health care: a convergent mixed-methods study in primary dental care
Source: Implement Sci. 2016 Jan 12;11:5. doi: 10.1186/s13012-015-0366-2 (PMC4710040; doi:10.1186/s13012-015-0366-2)
Supplement: Supplementary file 5 — Associated TDF domains identified in individual study components. [file 13012_2015_366_MOESM5_ESM.pdf]

Additional file 5
Associated TDF domains identified in individual study components

Additional file 5.1 – Diagnostic questionnaire, domain scales associated with best practice (p≤0.05)

**Record risk (N=60)**	**K**	**S**	**CA**	**CO**	**MI**	**MAD**	**E**	**SPI**	**SI**	**EM**	**BR**
Primary				✓	✓		✓	✓	✓		✓ *
Mixed			✓	✓	✓ *		✓	✓	✓		✓ *
Adult	✓			✓	✓		✓	✓	✓		✓ *
**Risk-based recall (N=73)**	**K**	**S**	**CA**	**CO**	**MI**	**MAD**	**E**	**SPI**	**SI**	**EM**	**BR**
Primary	✓		✓		✓	✓	✓				✓ *
Mixed	✓		✓	✓	✓	✓	✓ *		✓		
Adult											
**Apply fluoride varnish (N=60)**	**K**	**S**	**CA**	**CO**	**MI**	**MAD**	**E**	**SPI**	**SI**	**EM**	**BR**
Primary	✓			✓				✓	✓ *		
Mixed	✓			✓				✓ *			
Adult†	✓			✓	✓			✓ *	✓		
**Place fissure sealants (N=73)**	**K**	**S**	**CA**	**CO**	**MI**	**MAD**	**E**	**SPI**	**SI**	**EM**	**BR**
Primary†							✓				
Mixed											
Adult†											
**Demonstrate OH maint. (N=63)**	**K**	**S**	**CA**	**CO**	**MI**	**MAD**	**E**	**SPI**	**SI**	**EM**	**BR**
Primary											
Mixed					✓					✓	
Adult					✓				✓ *		
**Bitewing radiographs (N=63)**	**K**	**S**	**CA**	**CO**	**MI**	**MAD**	**E**	**SPI**	**SI**	**EM**	**BR**
Primary		✓			✓		✓			✓	
Mixed			✓ *	✓	✓ *				✓		
Adult									✓		

* Predictive of best practice in logistic regression (p≤0.05)
† No agreed guidance recommendations about best practice

K – knowledge; SPI – social professional role and identity; CA – beliefs about capabilities; CO – beliefs about consequences; MI – motivation, goals, and intent; MAD – memory, attention, and decision making; E – environmental context and resources; SI – social influence; EM – emotion; BR – behavioural regulation

Additional file 5
Associated TDF domains identified in individual study components

Additional file 5.2 – Dental practice case studies, domains associated with PMC behaviours

	K	S	CA	CO	MI	MAD	E	SPI	SI	EM	BR
**Record risk**	✓		✓	✓ *	✓		✓ *	✓	✓ *		✓
**Risk-based recall intervals**	✓		✓ *	✓ *			✓ *	✓			✓
**Apply fluoride varnish**	✓		✓	✓ *		✓	✓	✓ *	✓ *	✓	✓
**Place fissure sealants**	✓ *		✓	✓ *	✓			✓	✓ *		✓
**Demonstrate OH maintenance**	✓		✓	✓ *			✓ *	✓	✓ *	✓	✓
**Take bitewing radiographs**			✓	✓ *			✓	✓	✓ *		✓ *
**General preventive care**			✓	✓ *			✓ *	✓	✓ *	✓	✓

*Three most frequently coded domains for each behaviour

K – knowledge; SPI – social professional role and identity; CA – beliefs about capabilities; CO – beliefs about consequences; MI – motivation, goals, and intent; MAD – memory, attention, and decision making; E – environmental context and resources; SI – social influence; EM – emotion; BR – behavioural regulation

Additional file 5
Associated TDF domains identified in individual study components

Additional file 5.3 – Patient feedback, domains associated with PMC behaviours

**Apply fluoride varnish**	**K**	**S**	**CA**	**CO**	**MI**	**MAD**	**E**	**SPI**	**SI**	**EM**	**BR**
Adult†	✔ *										
Child	✔ *										
**Place fissure sealants**	**K**	**S**	**CA**	**CO**	**MI**	**MAD**	**E**	**SPI**	**SI**	**EM**	**BR**
Adult†											
Child											
**Demonstrate OH maintenance**	**K**	**S**	**CA**	**CO**	**MI**	**MAD**	**E**	**SPI**	**SI**	**EM**	**BR**
Adult				✔ *							
Child				✔ *							
**Take bitewing radiographs**	**K**	**S**	**CA**	**CO**	**MI**	**MAD**	**E**	**SPI**	**SI**	**EM**	**BR**
Adult											
Child											
**Oral health advice**	**K**	**S**	**CA**	**CO**	**MI**	**MAD**	**E**	**SPI**	**SI**	**EM**	**BR**
Adult			✔ *	✔ *			✔		✔ *		
Child	✔ *			✔ *			✔		✔ *	✔	
**Oral health maintenance**	**K**	**S**	**CA**	**CO**	**MI**	**MAD**	**E**	**SPI**	**SI**	**EM**	**BR**
Adult	✔		✔ *	✔ *	✔	✔	✔		✔ *	✔	
Child	✔		✔ *	✔ *	✔ *		✔		✔	✔	

* Three most frequently indexed domains for each behaviour
† No agreed guidance recommendations about best practice

K – knowledge; SPI – social professional role and identity; CA – beliefs about capabilities; CO – beliefs about consequences; MI – motivation, goals, and intent; MAD – memory, attention, and decision making; E – environmental context and resources; SI – social influence; EM – emotion; BR – behavioural regulation

Additional file 5
Associated TDF domains identified in individual study components

Additional file 5.4 – System level informant interviews, behavioural influences on PMC by COM-B

	Scottish Government		Postgraduate dental education		Professional association/Practice		Professional association	
**Capability**	**Facilitator**	**Barrier**	**Facilitator**	**Barrier**	**Facilitator**	**Barrier**	**Facilitator**	**Barrier**
Guidance	✓	✓	✓			✓		
Integrating prevention in					✓		✓	
Care planning					✓			
Oral health messages					✓			
Audit, Section 63			✓		✓			
Undergraduate training			✓					
Transition from training to								✓
**Opportunity**	**Facilitator**	**Barrier**	**Facilitator**	**Barrier**	**Facilitator**	**Barrier**	**Facilitator**	**Barrier**
Capacity				✓		✓		
Patient context						✓		✓
Environment						✓		✓
Data systems		✓			✓	✓		✓
Oral health initiatives	✓						✓	✓
Targeted fluoride varnish	✓	✓						
Dental care	✓				✓		✓	
Service division				✓		✓		✓
**Motivation**	**Facilitator**	**Barrier**	**Facilitator**	**Barrier**	**Facilitator**	**Barrier**	**Facilitator**	**Barrier**
Remuneration/finances	✓	✓		✓		✓		✓
Caries as priority issue				✓				
Prevention in general		✓			✓	✓		✓
