# Supplementary material for: Patient-, organization-, and system-level barriers and facilitators to preventive oral health care: a convergent mixed-methods study in primary dental care
Source: Implement Sci. 2016 Jan 12;11:5. doi: 10.1186/s13012-015-0366-2 (PMC4710040; doi:10.1186/s13012-015-0366-2)
Supplement: Supplementary file 6 — Salient domains matrix. Annotated domains influencing targeted PMC behaviours. [file 13012_2015_366_MOESM6_ESM.pdf]

Additional file 6
Salient domains matrix

COM-B	Capabilities				Opportunities		Motivation				
	Phy	Psychological			Social	Physical	Reflexive				Auto
**TDF**	**SK**	**Knowledge**	**MAD**	**Behavioural regulation**	**Social influence**	**Environment**	**Capabilities**	**Consequences**	**Social and professional role, identity**	**MI**	**EM**
**RECORD RISK**<br><br>Current practice:<br>**D: 82%**<br>**M: 84%**<br>**A: 48%**						S-D,M; CS<br>F – computerized records and recall systems; development and use of custom screens (CS)<br>F – time (S)<br>B/F – practice management (CS)<br>B – record and IT systems (CS)	S-D,M; CS<br>F – capabilities scale (not difficult; confident) (S)<br>B/F – record high risk but not low or moderate risk, know should record all risk levels but don't (CS)<br>B – uncertain in ability to ascribe risk (CS)<br>B – not confident ascribing risk (CS)	S-M; CS<br>F – improves quality of care (S)<br>B – no perceived benefit/point if cannot see pts in <6mo intervals (closely linked to RBR) (CS)			
**RISK BASED RECALL**<br><br>Current practice:<br>**D: 71%**<br>**M: 73%**<br>**A: 62%**		***Note:*** *Domain excluded as not an intervention target but lack of evidence base cited as key barrier*		S-All; CS<br>F – Usually do (S)<br>B/F – most use routine 6 mo recall, unable to tell if risk based or not due to NB (CS)	S-All; CS<br>F – supported by colleagues (S)<br>B – patients suspicious of shorter interval<br>B – patients only presenting in pain/problem (CS)	S-All; CS<br>F – environment scale (sufficient time; is not difficult to schedule) (S)<br>B –only reimbursed for 6mo recall interval (CS)<br>B/F – IT systems (CS)		S-All; CS<br>F – consequences scale (improves quality of care; improves patient OH) (S)<br>F – seeing pts frequently important even if not paid (CS)<br>B – no perceived benefit/point if not paid for <6mo interval in adults (CS)			
**APPLY FLUORIDE VARNISH**<br>Current practice:<br>**D: 81%**<br>**M: 67%**<br>A: 18%		S-All; CS; PFB<br>F – knowledge scale (supported by evidence; advocated in guidance) (S)<br>B – frequency of application (CS)<br>B – patients do not know about FV (PFB)			S-D,A ; CS<br>F – social influence scale (supported by colleagues; parents want for children) (S)<br>F – parents/pts request FV (CS)<br>F – Childsmile makes FV more familiar (CS)<br>B – parents/pts do not want (CS)<br>B – do not need to apply FV in GDP as done through Childsmile (CS)<br>B – parents/children do not attend Childsmile sessions, scheduling conflicts (CS)			S-All; CS<br>F – consequences scale (does not have negative consequences; benefits > costs; improves patient OH; prevents caries) (S)<br>F – belief that FC provides protection from decay (CS)<br>F – associated fee (CS)<br>B – perceived benefits insufficient to warrant use (CS)	S-All; CS<br>F – social and professional identity scale (important part of dentist's role; important part of dental team members' roles; dentist's responsibility) (S)<br>B – DCPs trained but do not have opportunity to do (CS)		

Current practice: Guidance recommended behaviours are in **bold**

Abbreviations: **A** – Adult dentition; **B** – Barrier; **BWR** – Bitewing radiographs; **CS** – Case studies; **D** – Deciduous dentition; **DCPs** – Dental care professionals; **DIT** – Data integration team; **DOHM** – Demonstration of oral health maintenance; **EM** – Emotion; **F** – Facilitator; **FS** – Fissure sealants; **FV** – Fluoride varnish; **GDP** – General dental practice; **IT** – Information technology; **M** – Mixed dentition; **MAD** – Memory, attention, decision making; **MI** – Motivation, goals, intent; **OHA** – Oral health advice; **PFB** – Patient feedback; **S** – Survey; **SK** – Skills

Additional file 6
Salient domains matrix

COM-B	Capabilities				Opportunity		Motivation				
	Phy	Psychological			Social	Physical	Reflexive				Auto
**TDF**	**SK**	**Knowledge**	**MAD**	**Behavioural regulation**	**Social influence**	**Environment**	**Capabilities**	**Consequences**	**Social and professional role, identity**	**MI**	**EM**
**PLACE PREVENTIVE FISSURE SEALANTS**<br>Current practice:<br>D: 16%<br>**M: 87%**<br>A: 23%					CS<br>F – parents/pts request FS (CS)			CS<br>F – associated fee (CS)<br>F – provide protection (CS)<br>B – concern that FS mask underlying decay (CS)			
**DEMONSTRATE OH MAINTENANCE**<br>Current practice:<br>**D: 87%**<br>**M: 91%**<br>**A: 71%**					S-A; CS<br>F – social influence scale (supported by colleagues; parents want for children; patients want) (S)<br>F – patients seek, actively request OHA (CS)<br>F – some patients in great need of OHA (CS)<br>B – DOHM, OHA makes patients feel patronized (CS) ***Note***: *Patients did not share this concern, typically wanted DOHM/OHA though some older adults did not think would influence their behaviour* (PFB)	CS<br>B – takes time which is not included/available as part of routine recall appointments (CS)<br>B – do not have models for demonstration (CS) ***Note:*** *Best practice recommendation is to demonstrate on patient, not a model*		CS; PFB<br>F – effective way to encourage good OH (CS)<br>F – benefits of showing > telling patient (CS, PFB)<br>B/F – benefits for children > adults (CS, PFB)	CS<br>F – DCPs consider part of their role (CS)<br>B – DCPs trained but do not have opportunity to do (CS)<br>B – dentists do not feel is their responsibility (CS)		
**BITEWING RADIOGRAPHS**<br>Current practice:<br>**D: 9%**<br>**M: 63%**<br>**A: 93%**					S-M,A; CS<br>F – social influences scale (parents want for children; patients want) (S)<br>B – cooperation from children (CS)<br>B – patients find BWR uncomfortable (CS)		S-M; CS<br>B – positioning films, quality of films (DIT)<br>B – difficult to do in children (deciduous dentition/young children hardest) (S, CS)	S-M; CS<br>F – consequences scale (does not have negative consequences for dentist; does not have negative consequences for patients) (S)<br>F – BWR show more than can be seen in clinical exam (CS)<br>B – in children and low risk adults, risk of radiation > benefit of BWR (CS)			

Current practice: Guidance recommended behaviours are in **bold**

Abbreviations: **A** – Adult dentition; **B** – Barrier; **BWR** – Bitewing radiographs; **CS** – Case studies; **D** – Deciduous dentition; **DCPs** – Dental care professionals; **DIT** – Data integration team; **DOHM** – Demonstration of oral health maintenance; **EM** – Emotion; **F** – Facilitator; **FS** – Fissure sealants; **FV** – Fluoride varnish; **GDP** – General dental practice; **IT** – Information technology; **M** – Mixed dentition; **MAD** – Memory, attention, decision making; **MI** – Motivation, goals, intent; **OHA** – Oral health advice; **PFB** – Patient feedback; **S** – Survey; **SK** – Skills

Additional file 6
Salient domains matrix

COM-B	Capabilities				Opportunity		Motivation				
	Phy	Psychological			Social	Physical	Reflexive				Auto
**TDF**	**SK**	**Knowledge**	**MAD**	**Behavioural regulation**	**Social influence**	**Environment**	**Capabilities**	**Consequences**	**Social and professional role, identity**	**MI**	**EM**
**ORAL HEALTH ADVICE**					CS; PFB<br>F – children especially receptive to advice (CS)<br>F – patients motivated to care for their OH (CS)<br>F – greater awareness in general of OH (CS)<br>F – colleagues, dental team members support preventive care (CS)<br>F – personalized, tailored approach (PFB)<br>F – caring, approachable manner in dentist (PFB)<br>F – dentist's OHA helpful, authoritative (PFB)<br>F – patients assume individual responsibility for OH maintenance; for children joint endeavour with dentist (PFB)<br>B – patient attitudes and beliefs limit receptivity to OHA, preventive care (diet/decay link, whether caries is preventable, prioritizing OH) (CS)<br>B – older patients are set in their ways (CS, PFB)<br>B – dentists' problem > prevention focus (PFB)<br>***Note:*** *Patients giving feedback all had reasonably good oral health maintenance practices. The patient level barriers cited in the CS may be patients with different levels of oral health maintenance.*	CS; PFB<br>F – customized computer screens (CS)<br>F – sufficient staff (CS)<br>F – sufficient resources (e.g. models, mirrors, printed information) (CS)<br>F – practice has an efficient system for recall and follow up care (CS)<br>F – practice is well managed, good communication among staff, time is made available for preventive care (CS)<br>F – dentists have time to answer questions (PFB)<br>F – dentists have free samples to try (PFB)<br>B – not paid for preventive care (CS)<br>B – insufficient space in practice (CS)<br>B – lack convenient time/day to offer Childsmile sessions (CS)	CS; PFB-A<br>F – have been trained to give preventive care and advice (CS)<br>F – provide a good service to patients (CS)<br>B/F – more recently trained (often younger) dentists give more preventive care and advice (PFB)<br>B – do not have enough training (CS)<br>B – feel lack communication skills necessary for preventive care (CS)	CS; PFB<br>F – purpose of dentistry is prevention, getting pts into habit of attending regularly will lead to better OH (CS)<br>F – OHA changes patient behaviour (PFB)<br>F – positive experiences will encourage children to keep attending (PFB)<br>B – not paid to do preventive care (CS)<br>B – OHA will not change OH of patients as too late to start prevention once problems established (CS)	CS<br>F – my responsibility to deliver general preventive care (DCPs and dentists) (CS)<br>F – patients expect dentist to give OHA/DOHM but would accept from DCPs (nurses) too (PFB)<br>F – patients open to OHA from multiple sources for children's OH in particular (PFB)<br>B –do not get opportunity to deliver preventive care (DCPs, nurses) (CS)<br>B – not my responsibility to give preventive care (dentists)(CS)		

Current practice: Guidance recommended behaviours are in **bold**

Abbreviations: **A** – Adult dentition; **B** – Barrier; **BWR** – Bitewing radiographs; **CS** – Case studies; **D** – Deciduous dentition; **DCPs** – Dental care professionals; **DIT** – Data integration team; **DOHM** – Demonstration of oral health maintenance; **EM** – Emotion; **F** – Facilitator; **FS** – Fissure sealants; **FV** – Fluoride varnish; **GDP** – General dental practice; **IT** – Information technology; **M** – Mixed dentition; **MAD** – Memory, attention, decision making; **MI** – Motivation, goals, intent; **OHA** – Oral health advice; **PFB** – Patient feedback; **S** – Survey; **SK** – Skills
